# Effects of exercise intervention on executive function in college students: a systematic review and meta-analysis of randomized controlled trials

**DOI:** 10.3389/fpubh.2026.1876948

**Published:** 2026-07-13

**Authors:** Wenhua Zhang, Keliang Chen, Jingqi Mei, Jun Xu

**Affiliations:** 1 College of Physical Education and Health, Guangxi Normal University, Guilin, Guangxi, China; 2 Department of Nursing, College of Medicine, Hunan Normal University, Changsha, Hunan, China

**Keywords:** college students, executive function, exercise intervention, meta-analysis, randomized controlled trial

## Abstract

**Objective:**

This study aims to systematically evaluate the effects of acute and long-term exercise interventions on executive functions (inhibitory control, working memory, cognitive flexibility) in college students, and to explore the moderating roles of measurement paradigms, exercise dosage, and scoring methods.

**Methods:**

We searched PubMed, Embase, Web of Science, Cochrane Library, Scopus, CNKI, and Wanfang from database inception to March 5, 2026, for randomized controlled trials on exercise interventions for executive function in college students. Two reviewers independently screened records, extracted data, and assessed risk of bias using the Cochrane ROB 2 tool. Meta-analyses were performed using Stata 17.0, with either fixed-effect or random-effect models selected based on heterogeneity.

**Results:**

A total of 16 randomized controlled trials involving 868 college students were included. Acute exercise interventions significantly improved inhibitory control (*g* = 0.52, 95% CI: 0.08, 0.95) and cognitive flexibility (*g* = 0.81, 95% CI: 0.23, 1.39), but the improvement in working memory did not reach statistical significance (*g* = 0.10, 95% CI: −0.21, 0.42). Long-term exercise interventions significantly improved inhibitory control (*g* = 0.52, 95% CI: 0.34, 0.70) and working memory (*g* = 0.57, 95% CI: 0.37, 0.78), whereas the effect size for cognitive flexibility was small and not statistically significant (*g* = 0.25, 95% CI: −0.05, 0.55). Subgroup analyses indicated that the effects of exercise interventions on subcomponents of executive function varied according to measurement paradigms, exercise dosage (duration, frequency, period, intensity), and scoring methods.

**Conclusion:**

Acute and long-term exercise show distinct patterns of improving executive functions in college students. Acute exercise significantly enhances cognitive flexibility and inhibitory control but has limited effects on working memory. Long-term exercise significantly enhances inhibitory control and working memory but shows no significant effect on cognitive flexibility.

**Systematic review registration:**

https://www.crd.york.ac.uk/, identifier CRD420261364227.

## 1 Introduction

Executive function is a set of higher-order cognitive processes that enable individuals to control their thoughts and actions in a goal-directed manner, typically comprising three core subcomponents: inhibitory control, working memory, and cognitive flexibility (1). Executive function plays an indispensable role in individuals’ academic achievement, daily life, and mental health. For college students, who are in a critical period of cognitive development, well-developed executive function is not only an important predictor of academic success but also closely related to personal life satisfaction and overall well-being (2).

However, college students face unique challenges. Research has shown that the transition from high school to university is often accompanied by increased academic pressure, lifestyle changes, and adaptation to new social environments, all of which may negatively affect students’ executive function (3). Meanwhile, problems such as sleep deprivation, sedentary behavior, and insufficient physical activity are prevalent among college students, further exacerbating executive function deficits (4). Impaired executive function can easily lead to various physical and mental health problems, including learning difficulties, attention deficits, anxiety, and depression (5). Therefore, exploring effective intervention strategies to improve executive function in college students has significant theoretical and practical implications.

In recent years, exercise intervention, as a safe, low-cost, non-pharmacological approach without drug side effects, has gained widespread attention in the field of cognitive function improvement. Research has confirmed that regular exercise not only enhances physical fitness but also positively affects brain structure and function, thereby improving executive function (6). Exercise interventions may promote executive function through multiple mechanisms, including increasing cerebral blood flow, promoting the release of neurotrophic factors such as brain-derived neurotrophic factor (BDNF), enhancing prefrontal cortex activity, and improving neuroplasticity (7). Different types of exercise (e.g., aerobic exercise, resistance training, high-intensity interval training, mind–body exercise) have all been shown to improve executive function to varying degrees (8). However, existing research findings are inconsistent, and there is considerable heterogeneity across studies in terms of exercise intervention protocols and executive function measurement paradigms, making it difficult to draw unified conclusions.

Several meta-analyses have examined the effects of exercise on executive function in young people. Haverkamp et al. (9) conducted a meta-analysis of the effects of physical activity interventions on cognitive outcomes in adolescents and young adults, reporting small-to-moderate effect sizes. Ludyga et al. (10) systematically reviewed moderators of long-term exercise effects on cognition in healthy individuals, but their analysis was not specifically focused on college students. Recently, Liu et al. (11) conducted a meta-analysis of exercise interventions for improving executive function in children with attention-deficit/hyperactivity disorder (ADHD), highlighting the critical influence of measurement paradigms and scoring methods—an issue that has received insufficient attention in healthy college populations. Furthermore, Ciria et al. (12) conducted an umbrella review of randomized controlled trials on the effects of physical exercise on cognition, calling for more rigorous methodologies. Singh et al. (6) provided a comprehensive umbrella review and meta-meta-analysis of exercise and cognition, but their focus broadly covered populations of different ages and health conditions rather than being specifically targeted at college students.

Despite these contributions, several research gaps remain. First, the neurobiological mechanisms of acute and long-term exercise are fundamentally different: acute exercise transiently enhances executive function by instantaneously activating the catecholamine system, increasing cerebral blood flow, and elevating cortical excitability (13, 14); whereas long-term exercise produces more persistent and cumulative cognitive benefits through mechanisms such as promoting BDNF expression, inducing structural remodeling of the hippocampus and prefrontal cortex, and enhancing synaptic plasticity (7, 15). However, existing meta-analyses have often combined acute and long-term exercise interventions in their analyses, which may obscure the true effect patterns of the two types of interventions on different subcomponents of executive function.

Second, no meta-analysis has systematically evaluated the effects of exercise interventions on executive functions in healthy college students while simultaneously reporting the effect sizes of acute and long-term exercise on each subcomponent separately. Third, the moderating roles of measurement paradigms and exercise dosage parameters (e.g., exercise type, intensity, frequency, period) on executive function outcomes in this population have not been comprehensively assessed. Fourth, previous meta-analyses have often combined different subcomponents of executive function (inhibitory control, working memory, cognitive flexibility) in their analyses without examining them separately, which may mask domain-specific effects.

Therefore, this systematic review and meta-analysis aims to systematically evaluate the effects of exercise interventions on executive functions in healthy college students, report separately the effect sizes of acute and long-term exercise on inhibitory control, working memory, and cognitive flexibility; explore the moderating roles of measurement paradigms, scoring methods, and exercise dosage parameters on intervention effects; and compare the differential effects and possible neural mechanisms of acute versus long-term exercise interventions, in order to provide evidence-based guidance for developing optimal exercise prescriptions for the college student population.

## 2 Materials and methods

### 2.1 Protocol and registration

This systematic review and meta-analysis followed the guidelines outlined in the Preferred Reporting Items for Systematic Reviews and Meta-Analyses (PRISMA) statement (16) (Supplementary Table S1). This study was registered on the PROSPERO platform with registration number: CRD420261364227.

### 2.2 Search strategy

A systematic literature search was conducted in PubMed, Embase, Web of Science, Cochrane Library, Scopus, China National Knowledge Infrastructure (CNKI), and Wanfang Database, covering the period from database inception to March 5, 2026. A combination of subject headings and free-text terms was used, covering three main conceptual domains: (1) exercise (e.g., sport, physical activity, aerobic exercise, resistance training, Wushu, Yoga, Taichi); (2) college students (e.g., college student, university student, undergraduate); (3) executive function (e.g., executive function, inhibitory control, working memory, cognitive flexibility). These three concept groups were combined using the Boolean operator “AND.” To ensure comprehensiveness, we manually searched grey literature, reference lists of included studies, trial registries (ClinicalTrials.gov, ChiCTR), and consulted domain experts. All retrieved records were imported into EndNote for literature management and subsequent screening. The complete search strategies for each database are presented in Supplementary Table S2.

### 2.3 Inclusion and exclusion criteria for the studies

The inclusion and exclusion criteria were developed based on the PICOS framework to enhance study credibility, reduce bias, and ensure robustness of the results.

Inclusion criteria: (1) The study must be a randomized controlled trial (RCT); (2) Participants must be generally healthy college students; (3) The study must investigate the effects of exercise or physical activity interventions on executive function in college students, with exercise as the primary intervention in the experimental group, while the control group only engaged in daily living or their usual physical activity without receiving additional exercise intervention; (4) Included studies must report outcome measures assessing executive function, including at least one of the three core subcomponents: inhibitory control, working memory, or cognitive flexibility; (5) Executive function assessment tools must have demonstrated reliability and validity; (6) There must be no significant baseline differences between the experimental and control groups prior to intervention; (7) Means and standard deviations for executive function outcomes in both groups must be directly available or derivable from the reported data.

Exclusion criteria: (1) Studies published in languages other than Chinese or English; (2) Studies involving participants with physical or neurological disorders that would prevent completion of the exercise intervention; (3) Studies in which the experimental group received non-exercise interventions or exercise combined with other interventions; (4) Reviews, meta-analyses, retrospective studies, observational studies, and cross-sectional studies; (5) Conference abstracts, dissertations, books, case reports, case series, and other descriptive or non-empirical studies; (6) Duplicate publications, retracted articles, studies with incomplete or inaccessible data, and studies with poor methodological quality.

### 2.4 Literature screening and data extraction

Retrieved records were imported into EndNote 20 software, and duplicates were removed. Subsequently, two researchers independently screened the literature and extracted information according to the predefined inclusion and exclusion criteria. Any disagreements were resolved through discussion with a third researcher. Characteristics of included studies included author, country of publication, study type, sample size, age, executive function measurement paradigm, outcome measures, and scoring method (Table 1). Exercise characteristics included exercise type, exercise intensity, exercise frequency, exercise duration, session time, and control group activities (Table 2). For studies with multiple experimental groups, only data from groups receiving exercise intervention alone were included.

**Table 1:** Characteristics of included studies.

Study	Country	Study type	Age (Years, T/C)	Sample size (T/C)	Outcome measure	Executive function measurement paradigm
Zhang (24) (2017)	China	RCT	19.36 ± 1.17	26/26	IC, WM, CF	Flanker, N-back, More-odd shifting
Eather (25) (2019)	Australia	RCT	20.23 ± 1.72/20.48 ± 2.01	26/27	CF	TMT
Li (26) (2020)	China	RCT	RE: 18.44 ± 0.51/AE: 18.43 ± 0.51/C: 18.58 ± 0.51	RE: 18/AE: 21/C: 19	IC, WM	Stroop, N-back
Su (27) (2021)	China	RCT	21.70 ± 2.55/23.37 ± 3.15	30/30	IC, WM, CF	Stroop, N-back, More-odd shifting
de Diego-Moreno (28) (2022)	Spain	RCT	MICT: 20.25 ± 1.23/HIIT: 21.62 ± 3.83/C: 21.35 ± 2.43	MICT: 28/HIIT: 27/C: 14	IC, WM	Stroop, N-back
Yang (29) (2023)	China	RCT	20 ± 2	HIIT: 20/MICT: 20/C: 20	IC, WM, CF	Flanker, N-back, More-odd shifting
Zhou (30) (2023)	China	RCT	20.0 ± 1.84	16/16	IC	Antisaccade
Wang (31) (2024)	China	RCT	20.08 ± 0.28/20.71 ± 0.51	35/35	IC	Stroop
Fan (32) (2025)	China	RCT	HIG: 22.05 ± 1.43/MIG: 21.20 ± 2.26/C: 21.15 ± 1.73	HIG: 20/MIG: 20/C: 20	IC, WM, CF	Stroop, Digit Span, WCST
Jimenez-Rolda (33) (2025)	Spain	RCT	18–25	HIIT: 21/HIIT+PA: 20/C: 17	IC, WM, CF	Stroop, Digit Span, WCST
Li (23)(2025)	China	RCT	18–21	33/33	IC, WM, CF	Flanker, N-back, More-odd shifting
Liu (34) (2025)	China	RCT	19.75 ± 1.24	32/32	IC, WM, CF	Stroop, N-back, More-odd shifting
Wallenwein (35) (2025)	Germany	RCT	22.5 ± 3.47/22.69 ± 2.51	32/26	IC, WM, CF	Go/No-Go, N-back, Task Switching
Curry (36) (2025)	USA	RCT	20.75 ± 2.56	LIG: 10/MIG: 10/HIG: 10\u00B0C: 10	CF	TMT
Si (17) (2025)	China	RCT	HIIT: 20.71 ± 1.36/MICT: 21.76 ± 1.79/C: 20.71 ± 1.36	17/17	IC, WM, CF	Stroop, N-back task, More-odd shifting
Xu (37) (2026)	China	RCT	20.12 ± 1.32/20.88 ± 3.28	17/17	IC	Stroop

T, treatment group; C, control group; RCT, randomized controlled trial; IC, inhibitory control; WM, working memory; CF, cognitive flexibility; TMT, Trail Making Test; WCST, Wisconsin Card Sorting Test; RE, resistance exercise; AE, aerobic exercise; MICT, moderate-intensity continuous training; HIIT, high-intensity interval training; HIG, high-intensity group; MIG, moderate-intensity group; LIG, light-intensity group.

**Table 2:** Exercise characteristics of included studies.

Study	Exercise type	Intervention duration	Exercise frequency	Session time	Exercise intensity	Control group activity
Zhang (24) (2017)	Running	8 weeks	3 times/week	30 min	60–69% HRmax	Normal study and life
Eather (25) (2019)	HIIT	8 weeks	3 times/week	8–12 min	≥85% HRmax	Continued usual physical activity
Li (26) (2020)	AE/RE	8 weeks	3 times/week	40 min	RE: 6–15 RM/AE: 60–69% HRmax	Maintained normal life and study
Su (27) (2021)	Cycle ergometer aerobic exercise	NR	1 time	20 min	60–70% HRmax	Reading materials
de Diego-Moreno (28) (2022)	HIFT/MICT	NR	1 time	30 min	HIFT: >85%HRmax/MICT: 70–80% HRmax	No exercise training
Yang (29) (2023)	HIIT/MICT	12 weeks	4 times/week	30 min	HIIT: ≥85% HRmax/MICT: 60–80% HRmax	Normal study and life
Zhou (30) (2023)	Cycle ergometer aerobic exercise	NR	1 time	15 min	60–70% HRmax	No exercise training
Wang (31) (2024)	24-form simplified Tai Chi	12 weeks	3 times/week	35 min	55%HRmax	Maintained original daily routine
Fan (32) (2025)	Cycle ergometer aerobic exercise HIG/MIG	NR	1 time	20 min	HIG: 70-85%HRmax/MIG: 50-70%HRmax	Sitting and resting
Jimenez-Rolda (33) (2025)	HIIT/HIIT+PA (multimodal exercise)	12 weeks	3 times/week	25 min	HIIT: CR-10 8–10	No structured exercise
Li (23) (2025)	Tai Chi	8 weeks	3 times/week	40 min	NR	Maintained normal physical activity level
Liu (34) (2025)	Running	8 weeks	3 times/week	30 min	60–69% HRmax	No exercise intervention
Wallenwein (35) (2025)	Jump training	8 weeks	3 times/week	15 min	High intensity (specific HR or RPE not quantified)	Maintained regular routine
Curry (36) (2025)	Cycle ergometer aerobic exercise LIG/MIG/HIG	NR	1 time	12 min	LIG: 35–40% HRR/MIG: 55–60% HRR/HIG: 75–80% HRR	No exercise
Xu (37) (2026)	Tabata training	NR	1 time	4 min	High intensity (Borg CR-10 score: 8.68/10)	No exercise

RE, resistance exercise; AE, aerobic exercise; MICT, moderate-intensity continuous training; HIIT, high-intensity interval training; HIG, high-intensity group; MIG, moderate-intensity group; LIG, light-intensity group; PA, physical activity; HRmax, heart rate maximum; RM, repetition maximum; RPE, rate of perceived exertion; HRR, heart rate reserve; CR, category-ratio, NR, not report.

Because the included studies used different scoring methods for executive function outcomes (positive scoring: higher values indicate better function; reverse scoring: lower values indicate better function), we standardized the effect direction so that positive values consistently indicate improvement. For positively scored indicators (e.g., accuracy), we converted them to reverse-scored indicators (error rate = 1 − accuracy) and recalculated standard deviations using the variance propagation formula (17). For originally reverse-scored indicators (e.g., reaction time, error rate), we retained their original values. We then multiplied all reverse-scored effect sizes by −1, ensuring that positive pooled effect sizes always represent improvement. When a study reported multiple indicators, we prioritized the most commonly used indicator in the field; if no priority could be determined, we averaged the effect sizes.

(1) Mean conversion

$${\text{Mean}}_{\text{error}}=1-{\text{Mean}}_{\text{accuracy}}$$

(2) Standard deviation conversion (according to the variance propagation formula)

$${\mathrm{SD}}_{\text{error}}={\mathrm{SD}}_{\text{accuracy}}$$

For multi-arm trials sharing a common control group, we used the split-control-group correction method to avoid double counting. The sample size of the shared control group was equally divided according to the number of experimental arms, while the mean and standard deviation of the control group remained unchanged (18). This approach followed the Cochrane Handbook’s recommendations for handling multi-arm trials.

### 2.5 Risk of bias assessment

Two independent reviewers assessed the risk of bias of the included studies using the revised Cochrane Risk of Bias Tool for Randomized Trials (ROB 2), evaluating the following five domains: randomization process, deviations from intended interventions, missing outcome data, measurement of the outcome, and selective reporting of results. According to the corresponding algorithms, each domain was judged as “low risk,” “some concerns,” or “high risk.” After learning the Cochrane Risk of Bias Tool and conducting pilot assessments, the two independent reviewers performed risk of bias assessments and subsequently cross-checked their results. Any disagreements were resolved through discussion between the two reviewers or by consulting a third reviewer.

### 2.6 Data analysis

All statistical analyses were performed using Stata 17. Due to differences in outcome measurements across studies, the standardized mean difference ( *SMD* ) and its 95% confidence interval (CI) were calculated. Hedges‘g was used to correct for small sample bias. Heterogeneity among studies was assessed using the chi-square test and *I*^2^ statistic. Low heterogeneity was defined as *p* > 0.1 and *I*^2^ < 50%, in which case a fixed-effect model was used. Significant heterogeneity (*p* < 0.1 and *I*^2^ > 50%) warranted the use of a random-effect model (19). Effect sizes were interpreted as follows: 0.2 ≤ *d* < 0.5 indicated a small effect, 0.5 ≤ *d* ≤ 0.8 indicated a moderate effect, and *d* > 0.8 indicated a large effect.

### 2.7 Subgroup analysis

Based on the published literature, subgroup analyses were conducted using different executive function measurement paradigms, exercise intensity, exercise frequency, session duration, and exercise duration as moderators to identify sources of heterogeneity. Exercise intensity was classified according to the standards outlined in the American College of Sports Medicine (ACSM) *Guidelines for Exercise Testing and Prescription* (2018) (20), with detailed classification criteria presented in Supplementary Table S3 of the Supplementary materials. Additionally, exploratory subgroup analyses were conducted based on the primary type of exercise (e.g., aerobic exercise, high-intensity interval training, resistance training, Tai Chi, etc.) to compare the effects of different exercise modalities on executive function. Given the limited number of studies in certain categories, these analyses are hypothesis-generating and should be interpreted with caution. The results are presented in the Supplementary materials.

### 2.8 Publication bias assessment

According to the Cochrane Handbook recommendations, funnel plots and Egger’s tests were used to assess publication bias only when the number of studies included for a given outcome measure was 10 or more (21, 22). For subgroups with fewer than 10 included studies, formal tests for publication bias were not performed due to insufficient statistical power.

### 2.9 Sensitivity analysis

Sensitivity analysis was performed using two approaches. First, the leave-one-out method was used, systematically excluding one study at a time, to assess the robustness of the results. Second, to evaluate the potential impact of ambiguous exercise intensity classification, we conducted an additional sensitivity analysis by excluding studies that did not clearly report exercise intensity (i.e., those marked as “NR” in Table 2) (23). For outcomes involving these studies, the pooled effect sizes were recalculated after exclusion, and the results before and after exclusion were compared.

## 3 Results

### 3.1 Study selection

**Figure 1:**
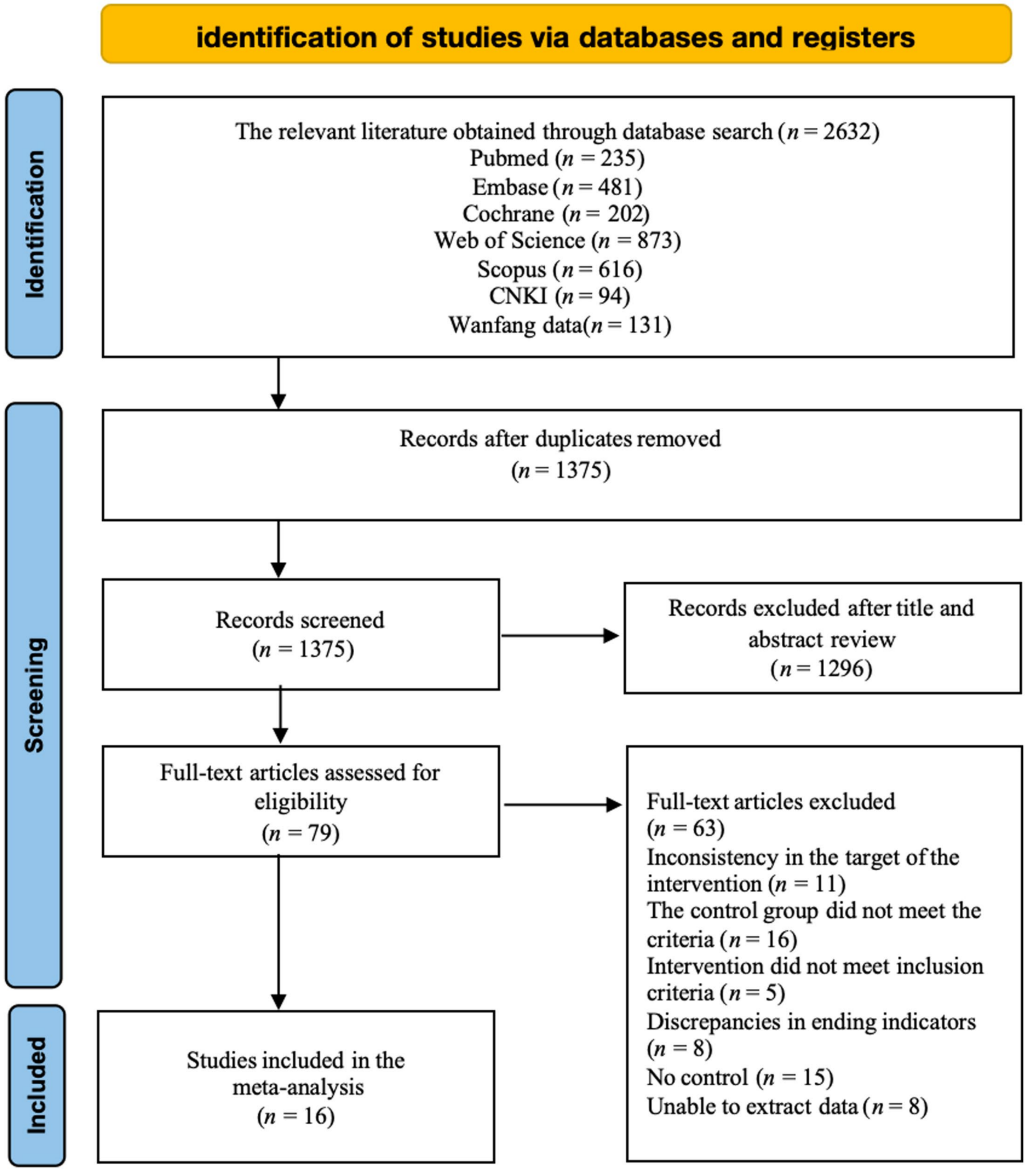
PRISMA study flow diagram.

A total of 2,632 records were retrieved from PubMed, Embase, Web of Science, Cochrane Library, Scopus, CNKI, and Wanfang Database. After screening by title, abstract, and full text according to the inclusion and exclusion criteria, 16 studies (17, 23–37) were finally included in the meta-analysis. The screening process is shown in Figure 1.

### 3.2 Characteristics of the included studies

A total of 16 studies (17, 23–37) published between 2017 and 2026 were included in this meta-analysis, with a total of 868 participants (509 in the experimental group and 359 in the control group). Regarding country of publication, 11 studies (17, 23, 24, 26, 27, 29–32, 34, 37) were from China, 2 from Spain (28, 33), 1 from Australia (25), 1 from Germany (35), and 1 from the United States (36) (Table 1). Among the included studies, 14 studies (17, 23, 24, 26–35, 37) evaluated the effects of exercise interventions on inhibitory control in college students, 10 studies (17, 23, 24, 26–29, 32, 34, 35) evaluated working memory, and 10 studies (23–25, 27, 29, 32–36) evaluated cognitive flexibility (Table 1). The exercise interventions implemented in the experimental groups included high-intensity interval training, aerobic exercise, resistance training, jumping exercise, Tai Chi, and Tabata training (Table 2). The intervention duration ranged from 6 to 12 weeks; specifically, 3 studies (29, 31, 33) implemented a 12-week protocol, 6 studies (23–26, 34, 35) used an 8-week intervention, Only one study (17) employed a 6-week intervention, and 6 studies (27, 28, 30, 32, 36, 37) investigated the effects of a single acute bout of exercise (Table 2). The intervention frequency was 3–4 sessions per week (Table 2). Session duration ranged from 4 to 60 min (Table 2). Regarding exercise intensity, 10 studies (17, 24, 26–30, 32, 34, 36) reported moderate-intensity exercise, 9 studies (17, 25, 28, 29, 32, 33, 35–37) reported high-intensity exercise, 2 studies (31, 36) reported low-intensity exercise, and 1 study (23) did not report exercise intensity (Table 2).

### 3.3 Risk of bias

**Figure 2:**
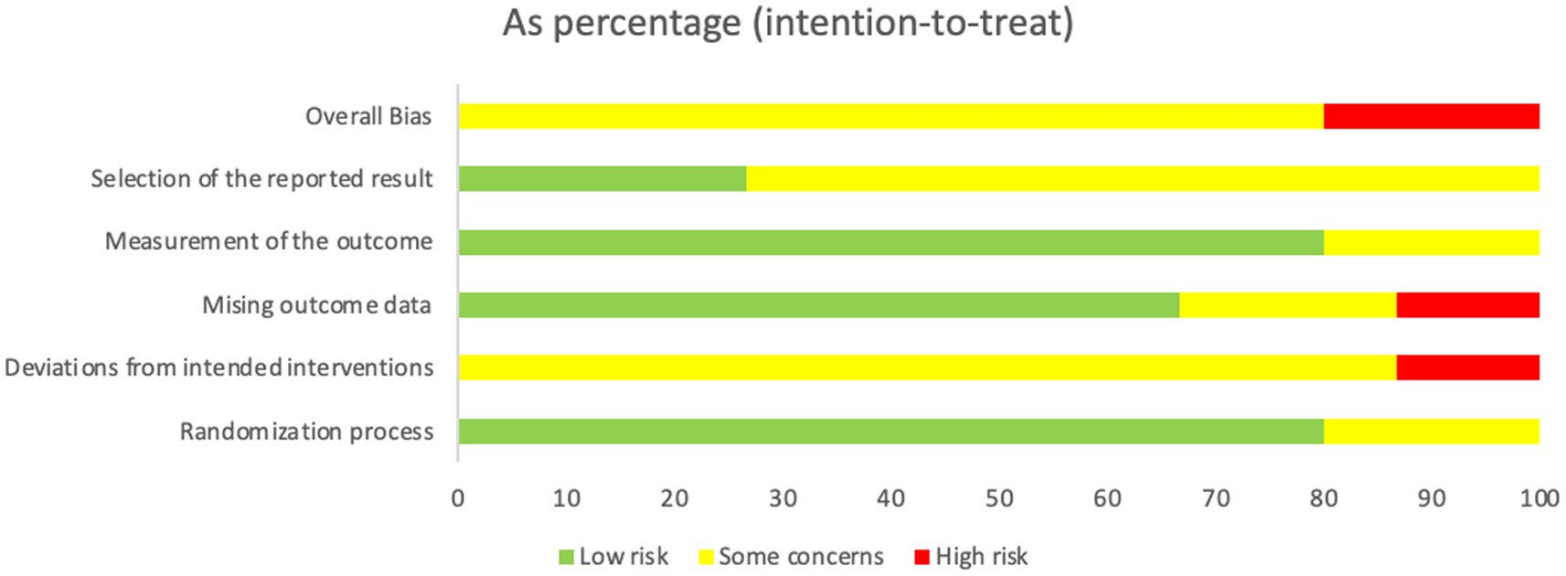
Risk of bias of the included studies.

**Figure 3:**
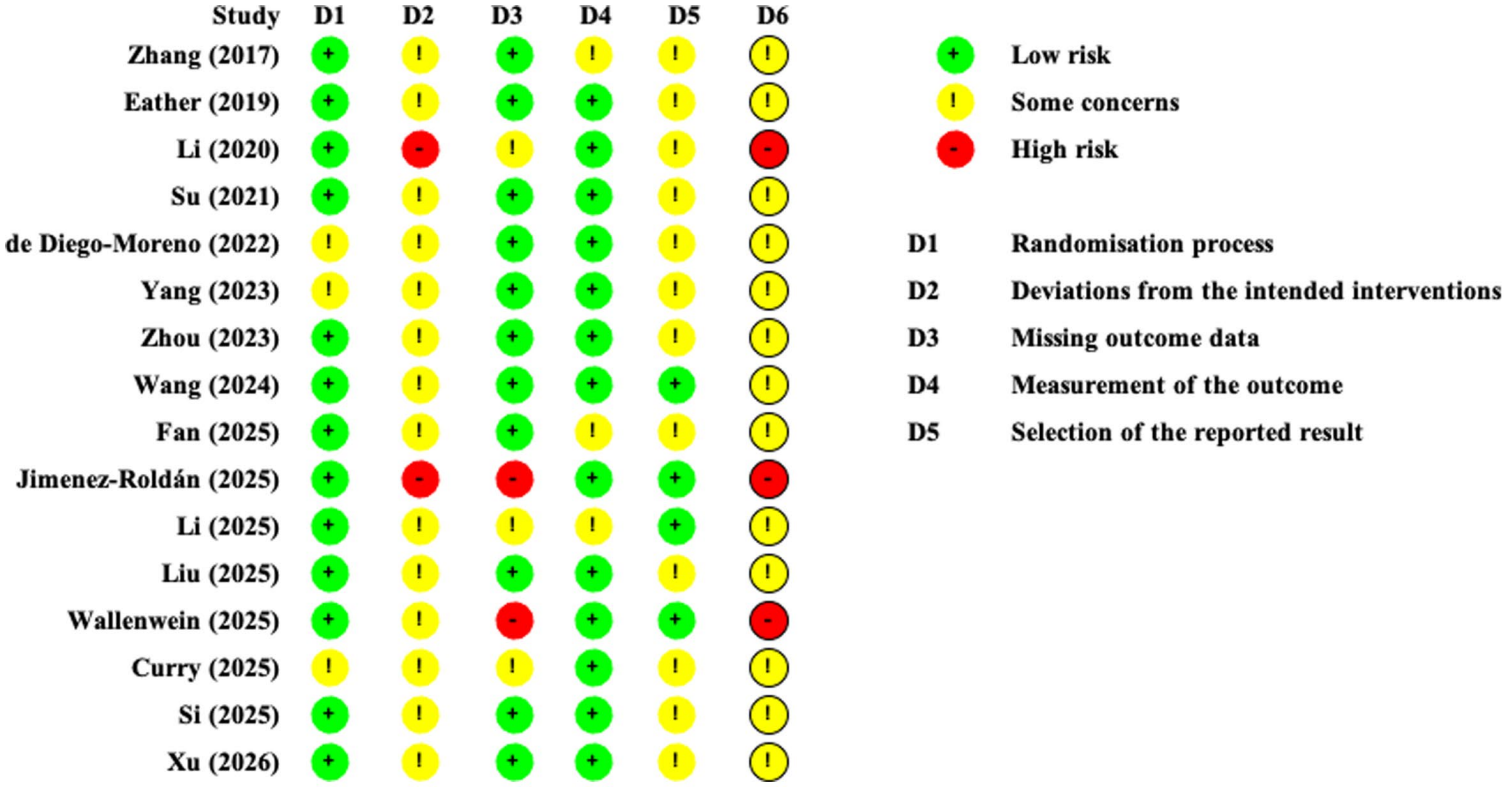
Risk of bias summary of the included studies.

Among the 15 included RCTs, 11 studies (23–27, 30–35, 37) reported specific methods of randomization, primarily including computer-generated random sequences, block randomization, or stratified randomization; only 3 studies (23, 25, 31) explicitly mentioned specific measures for allocation concealment, such as using sealed opaque envelopes or having group assignment performed by an independent third party; 4 studies (23, 25, 33, 35) clearly reported blinding of outcome assessors, but due to the nature of exercise interventions, participants could not be blinded to the intervention. Ten studies (23, 25, 26, 30, 32–37) reported dropouts and losses to follow-up. Regarding analysis principles, only 2 studies (23, 33) explicitly stated that they followed the intention-to-treat (ITT) analysis, while some studies used per-protocol (PP) analysis or did not clearly specify the analysis method. Only 5 studies (23, 25, 31, 33, 34) provided clinical trial registration numbers. The results of the risk of bias assessment are shown in Figures 2, 3.

### 3.4 Meta-analysis results

After harmonizing the direction of effect sizes so that positive values consistently indicate improvement in executive function (i.e., effect sizes for reverse-scored outcomes were multiplied by −1), meta-analyses were performed separately for inhibitory control, working memory, and cognitive flexibility.

#### 3.4.1 Effects of acute exercise interventions on executive function

**Figure 4:**
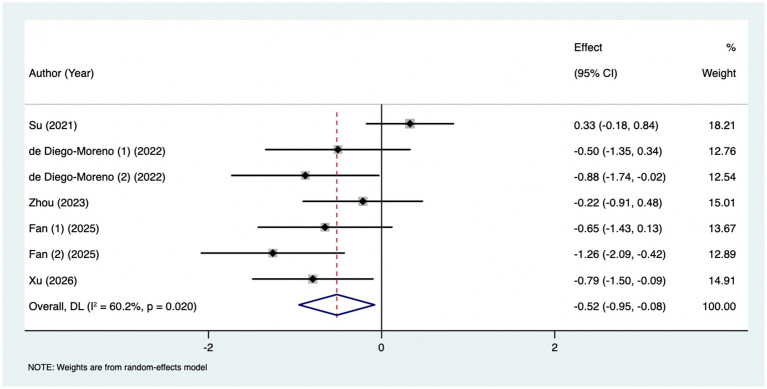
Forest plot of the effects of acute exercise interventions on inhibitory control.

**Figure 5:**
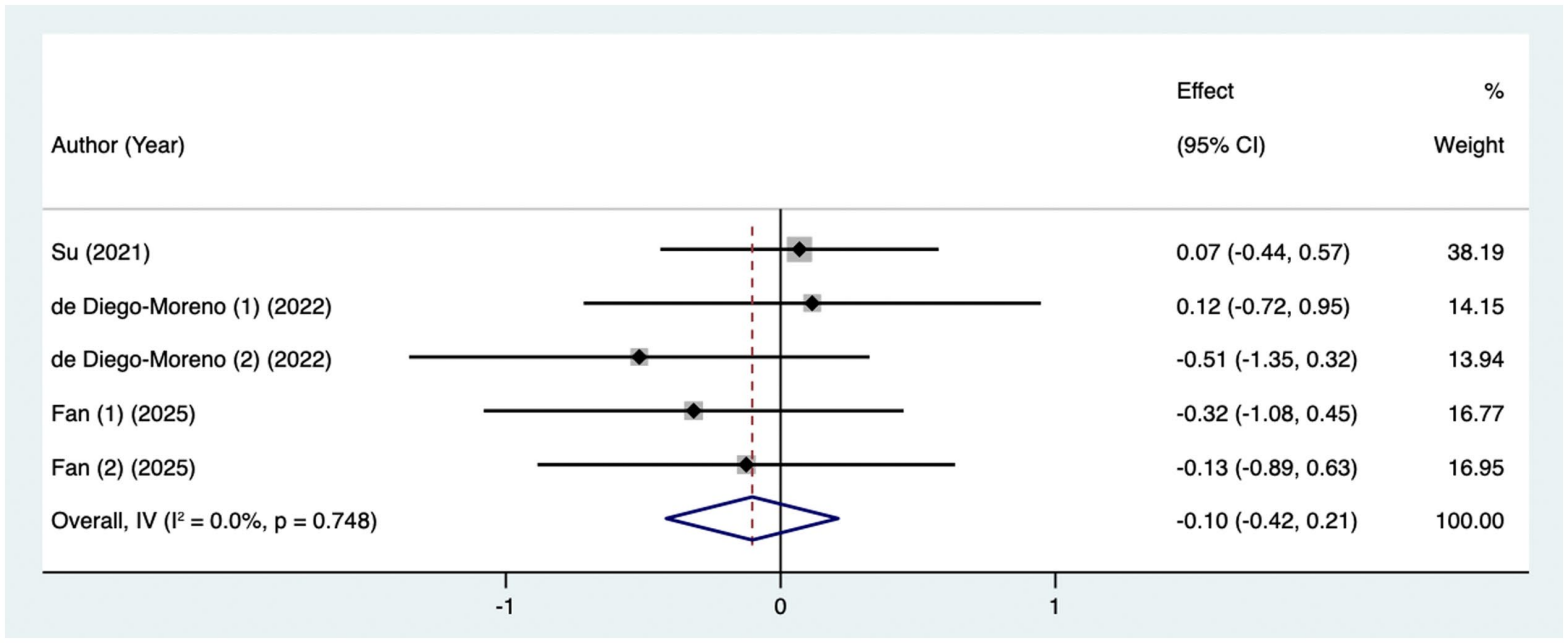
Forest plot of the effects of acute exercise interventions on working memory.

**Figure 6:**
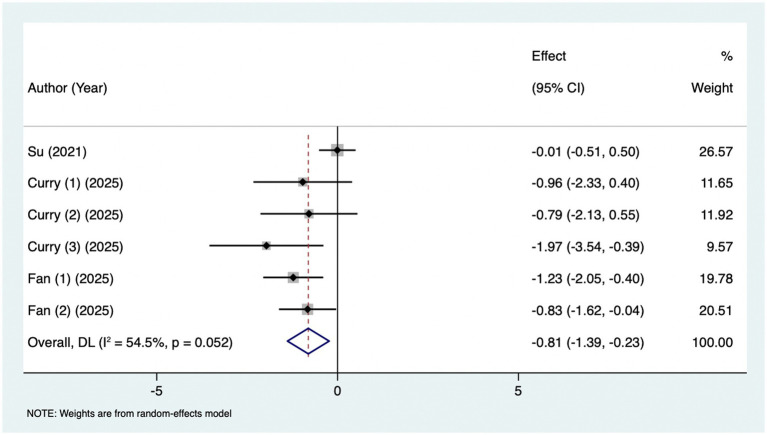
Forest plot of the effects of acute exercise interventions on cognitive flexibility.

For inhibitory control, a total of 5 studies were included. Due to significant heterogeneity among studies (*I*^2^ = 60.2%, *p* = 0.020), a random-effects model was used. The overall effect of acute exercise interventions on inhibitory control in college students was significant (*g* = 0.52, 95% CI: 0.08, 0.95) (Figure 4). For working memory, a total of 3 studies were included. There was no heterogeneity among studies (*I*^2^ = 0.0%, *p* = 0.748), and a fixed-effect model was used. The overall effect of acute exercise interventions on working memory in college students was not statistically significant (*g* = 0.10, 95% CI: −0.21, 0.42) (Figure 5). For cognitive flexibility, a total of 3 studies were included. Moderate heterogeneity was observed among studies (*I*^2^ = 54.5%, *p* = 0.052), and a random-effects model was used. The overall effect of acute exercise interventions on cognitive flexibility in college students was significant (*g* = 0.81, 95% CI: 0.23, 1.39) (Figure 6).

#### 3.4.2 Effects of long-term exercise interventions on executive function

**Figure 7:**
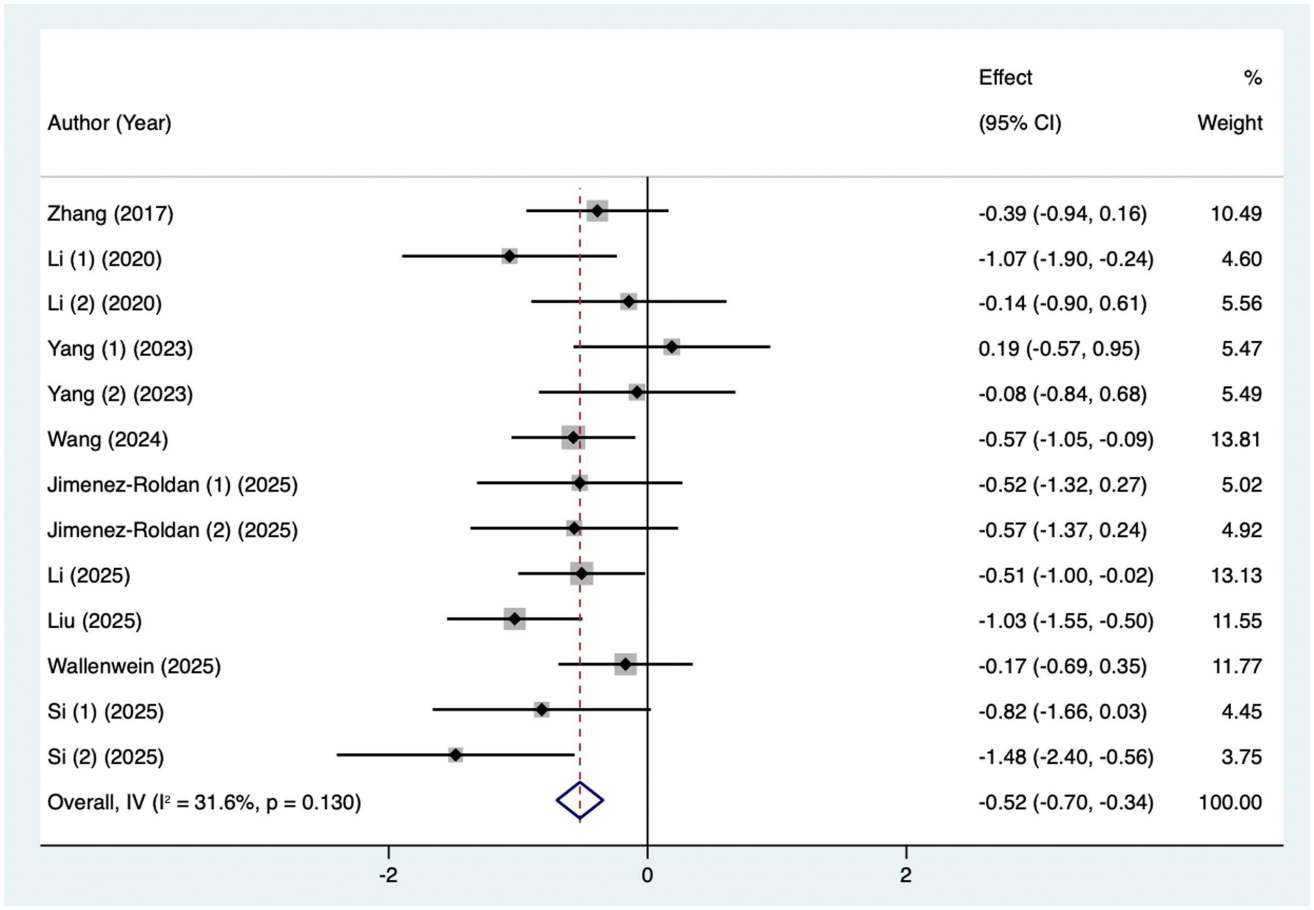
Forest plot of the effects of long-term exercise interventions on inhibitory control.

**Figure 8:**
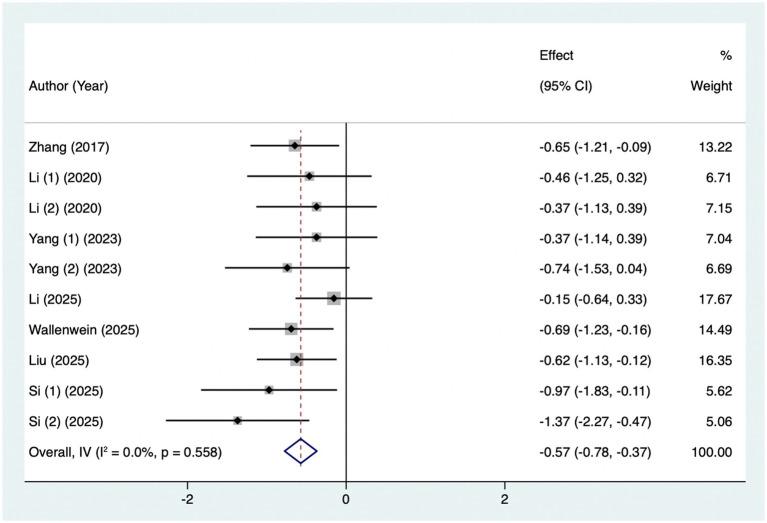
Forest plot of the effects of long-term exercise interventions on working memory.

**Figure 9:**
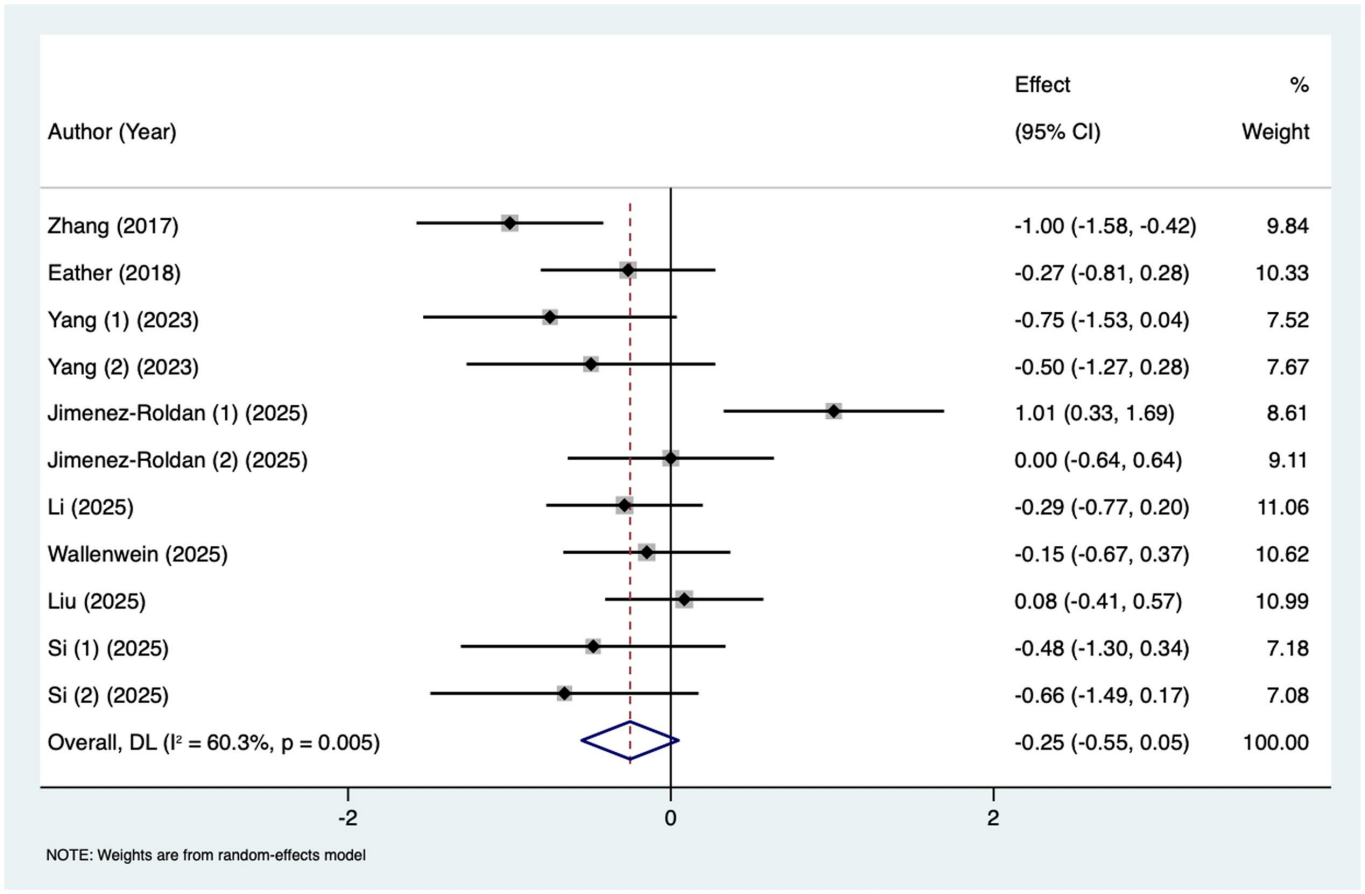
Forest plot of the effects of long-term exercise interventions on cognitive flexibility.

For inhibitory control, a total of 9 studies were included. Due to low heterogeneity among studies (*I*^2^ = 31.6%, *p* = 0.130), a fixed-effect model was used. The overall effect of long-term exercise interventions on inhibitory control in college students was significant (*g* = 0.52, 95% CI: 0.34, 0.70) (Figure 7). For working memory, a total of 7 studies were included. There was no heterogeneity among studies (*I*^2^ = 0.0%, *p* = 0.558), and a fixed-effect model was used. The overall effect of long-term exercise interventions on working memory in college students was significant (*g* = 0.57, 95% CI: 0.37, 0.78) (Figure 8). For cognitive flexibility, a total of 8 studies were included. Significant heterogeneity was observed among studies (*I*^2^ = 60.3%, *p* = 0.005), and a random-effects model was used. The overall effect of long-term exercise interventions on cognitive flexibility in college students was not statistically significant (*g* = 0.25, 95% CI: −0.05, 0.55) (Figure 9).

### 3.5 Subgroup analysis

#### 3.5.1 Subgroup analysis of acute exercise interventions on inhibitory control

The results of subgroup analyses for acute exercise interventions on inhibitory control are presented in Supplementary Table S4. Regarding measurement paradigms, the Stroop color-word test (*g* = 0.41, 95% CI: −0.23, 1.05), Go/No-Go task (*g* = 0.94, 95% CI: 0.35, 1.53), and antisaccade task (*g* = 0.22, 95% CI: −0.48, 0.91) all showed positive effects, but only the Go/No-Go task reached statistical significance. In terms of exercise type, aerobic exercise (*g* = 0.48, 95% CI: −0.11, 1.07), high-intensity functional training (*g* = 0.50, 95% CI: −0.34, 1.35), and Tabata training (*g* = 0.79, 95% CI: 0.09, 1.50) all showed beneficial effects, but only Tabata training reached statistical significance. Regarding exercise intensity, high-intensity exercise (*g* = 0.67, 95% CI: 0.23, 1.11) had a larger effect size than light-to-moderate intensity exercise (*g* = 0.45, 95% CI: −0.27, 1.18), and high-intensity exercise reached statistical significance.

#### 3.5.2 Subgroup analysis of long-term exercise interventions on inhibitory control

The results of subgroup analyses for long-term exercise interventions on inhibitory control are presented in Supplementary Table S5. Regarding measurement paradigms, the Stroop color-word test (*g* = 0.75, 95% CI: 0.50, 0.99) and Flanker task (*g* = 0.29, 95% CI: −0.01, 0.60) both showed improvement effects, with the Stroop color-word test reaching statistical significance. In terms of exercise type, aerobic exercise (*g* = 0.51, 95% CI: 0.24, 0.79), resistance training (*g* = 1.07, 95% CI: 0.24, 1.90), high-intensity interval training (*g* = 0.66, 95% CI: 0.08, 1.24), Tai Chi (*g* = *g* = 0.54, 95% CI: 0.20, 0.88), and multimodal exercise (*g* = 0.57, 95% CI: −0.24, 1.37) all showed improvement effects, among which aerobic exercise, resistance training, high-intensity interval training, and Tai Chi reached statistical significance. Regarding session duration, the effect size for ≥30 min (*g* = 0.59, 95% CI: 0.37, 0.81) was larger than that for <30 min (*g* = 0.40, 95% CI: 0.10, 0.71). Regarding exercise intensity, both light-to-moderate intensity (*g* = 0.62, 95% CI: 0.38, 0.86) and high intensity (*g* = 0.36, 95% CI: 0.05, 0.68) showed significant improvement. Regarding intervention duration, 6-week (*g* = 1.12, 95% CI: 0.50, 1.74), 8-week (*g* = 0.53, 95% CI: 0.30, 0.77), and 12-week (*g* = 0.37, 95% CI: 0.07, 0.67) interventions all showed significant improvement, with the 6-week intervention having the largest effect size.

#### 3.5.3 Subgroup analysis of acute exercise interventions on working memory

The results of subgroup analyses for acute exercise interventions on working memory are presented in Supplementary Table S6. In terms of exercise type, neither aerobic exercise (*g* = 0.14, 95% CI: −0.20, 0.48) nor high-intensity functional training (*g* = −0.12, 95% CI: −0.95, 0.72) reached statistical significance. Regarding exercise intensity, neither light-to-moderate intensity (*g* = 0.10, 95% CI: −0.28, 0.47) nor high intensity (*g* = *g* = 0.12, 95% CI: −0.44, 0.68) showed significant improvement effects.

#### 3.5.4 Subgroup analysis of long-term exercise interventions on working memory

The results of subgroup analyses for long-term exercise interventions on working memory are presented in Supplementary Table S7. In terms of exercise type, aerobic exercise (*g* = 0.69, 95% CI: 0.40, 0.98), high-intensity interval training (*g* = 0.64, 95% CI: 0.07, 1.21), resistance training (*g* = 0.79, 95% CI: 0.32, 1.25), and jump training (*g* = 0.69, 95% CI: 0.16, 1.23) all showed significant improvement effects, whereas Tai Chi (*g* = 0.15, 95% CI: −0.33, 0.64) did not reach statistical significance. Regarding session duration, the effect size for ≥30 min (*g* = 0.55, 95% CI: 0.33, 0.77) was smaller than that for <30 min (*g* = *g* = 0.69, 95% CI: 0.16, 1.23). In terms of exercise intensity, both light-to-moderate intensity (*g* = 0.66, 95% CI: 0.39, 0.93) and high intensity (*g* = 0.67, 95% CI: 0.28, 1.06) showed significant improvement effects. Regarding intervention duration, 6-week (*g* = 1.16, 95% CI: 0.54, 1.78), 8-week (*g* = 0.49, 95% CI: 0.26, 0.73), and 12-week (*g* = 0.55, 95% CI: 0.00, 1.10) interventions all showed significant improvement, with the 6-week intervention having the largest effect size.

#### 3.5.5 Subgroup analysis of acute exercise interventions on cognitive flexibility

The results of subgroup analyses for acute exercise interventions on cognitive flexibility are presented in Supplementary Table S8. Regarding measurement paradigms, both the More-odd shifting task (*g* = *g* = 0.63, 95% CI: 0.14, 1.40) and the Trail Making Test (*g* = 1.17, 95% CI: 0.35, 1.99) showed improvement effects, and both reached statistical significance. In terms of exercise intensity, high-intensity exercise (*g* = 1.16, 95% CI: 0.45, 1.86) had a larger effect size than light-to-moderate intensity exercise (*g* = 0.69, 95% CI: −0.06, 1.44), and high-intensity exercise reached statistical significance.

#### 3.5.6 Subgroup analysis of exercise interventions on cognitive flexibility under reverse scoring

The results of subgroup analyses for long-term exercise interventions on cognitive flexibility are presented in Supplementary Table S9. Regarding measurement paradigms, the More-odd shifting task (*g* = 0.46, 95% CI: 0.16, 0.77) showed a significant improvement effect, whereas the Wisconsin Card Sorting Test (*g* = −0.50, 95% CI: −1.49, 0.49), the Trail Making Test (*g* = 0.27, 95% CI: −0.28, 0.81), and task-switching paradigms (*g* = 0.15, 95% CI: −0.37, 0.67) did not reach statistical significance. In terms of exercise type, aerobic exercise (*g* = 0.39, 95% CI: −0.06, 0.84) showed a certain trend toward improvement but did not reach statistical significance, whereas high-intensity interval training (*g* = 0.11, 95% CI: −0.65, 0.86, *I*^2^ = 78.5%), jump training (*g* = 0.29, 95% CI: −0.20, 0.77), and Tai Chi (*g* = 0.15, 95% CI: −0.37, 0.67) showed no significant effects. Regarding session duration, the effect size for ≥30 min (*g* = 0.46, 95% CI: 0.16, 0.77) was significantly larger than that for <30 min (*g* = −0.12, 95% CI: −0.64, 0.41). In terms of exercise intensity, neither light-to-moderate intensity (*g* = 0.49, 95% CI: −0.05, 1.03) nor high intensity (*g* = 0.09, 95% CI: −0.35, 0.53) reached statistical significance. Regarding intervention duration, 6-week (*g* = 0.57, 95% CI: −0.02, 1.15) and 8-week (*g* = 0.30, 95% CI: −0.03, 0.64,) interventions showed a certain trend toward improvement, but neither reached statistical significance; the 12-week intervention (*g* = 0.04, 95% CI: −0.72, 0.80) showed no improvement effect.

### 3.6 Publication bias

In the long-term exercise subgroup, the number of included studies for inhibitory control (*n* = 9), working memory (*n* = 7), and cognitive flexibility (*n* = 8) did not reach 10 for any outcome; therefore, statistical power was insufficient, and formal tests for publication bias were not performed. In the acute exercise subgroup, the number of included studies was even smaller (*n* = 3–5), and neither funnel plots nor Egger’ s tests were conducted.

### 3.7 Sensitivity analysis

After sequentially excluding each study, the effect sizes all fell within the 95% confidence intervals of the overall effect size, indicating that the impact on the pooled effect size was small and acceptable. This enhances the robustness of the original meta-analysis results, making them more convincing (Supplementary Figures S1–S6).

Furthermore, we performed a sensitivity analysis excluding the study that did not report exercise intensity (23). For long-term inhibitory control, the pooled effect size changed from (*g* = 0.52, 95% CI: 0.34, 0.70) to (*g* = 0.54, 95% CI: 0.34, 0.73) after exclusion, with heterogeneity decreasing from *I*^2^ = 31.6% to *I*^2^ = 40.4% (Supplementary Figure S7). For long-term working memory, the pooled effect size changed from (*g* = 0.57, 95% CI: 0.37, 0.78) to (*g* = 0.68, 95% CI: 0.45, 0.90) after exclusion, with heterogeneity remaining low (*I*^2^ = 0.0%) (Supplementary Figure S8). For long-term cognitive flexibility, the pooled effect size changed from (*g* = 0.25, 95% CI: −0.05, 0.55) to (*g* = 0.26, 95% CI: −0.09, 0.61) after exclusion, with heterogeneity remaining substantial (*I*^2^ = 65.7%) (Supplementary Figure S9). Notably, the statistical significance of each outcome remained unchanged (inhibitory control and working memory remained significant, cognitive flexibility remained non-significant), confirming that the inclusion of the study with unreported exercise intensity did not materially affect the overall conclusions.

## 4 Discussion

### 4.1 Effects of exercise interventions on inhibitory control in college students

This study found that both acute and long-term exercise significantly improve college students’ inhibitory control, with the effect sizes being highly comparable between the two. In recent years, accumulating neuroimaging evidence has provided a solid biological basis for this finding. An activation likelihood estimation (ALE) meta-analysis including 20 functional magnetic resonance imaging studies showed that exercise interventions significantly altered brain activation patterns during executive function tasks in healthy populations, particularly in the frontal lobe, precuneus, thalamus, and cingulate gyrus regions (38). Remiszewski et al. (39) conducted a 12-week RCT using a modified Flanker task combined with behavioral assessments to evaluate the effects of cardiovascular exercise on inhibitory control in sedentary young adults. The study found that the experimental group showed progressive reductions in reaction time during incongruent trials without compromising accuracy, whereas although the control group also showed reduced reaction time, overall accuracy decreased, suggesting that exercise interventions specifically improve inhibitory control efficiency. Wu et al. (40) found in a 12-week exercise intervention study that exercise interventions induced different patterns of neuroplasticity at different stages: the first 6 weeks primarily increased activation in the right superior medial frontal gyrus, while the latter 6 weeks showed decreased activation in the left superior medial frontal gyrus. This dynamic pattern of neural adaptation suggests that the improvement of inhibitory control through exercise may involve neural resource reorganization mechanisms (40).

Subgroup analysis showed that exercise dosage is an important moderating variable affecting the improvement of inhibitory control. A systematic review and meta-analysis of children and adolescents found that acute exercise interventions had significant effects on inhibitory control, indicating that even a single bout of acute exercise can positively affect inhibitory control (41). Liu et al. (3) used a longitudinal experimental design and found that reallocating 30 min of daily sedentary time to light physical activity significantly reduced Trail Making Test completion time, suggesting improved cognitive processing speed; reallocating the same duration to moderate-to-vigorous physical activity improved task accuracy. The study also observed a dose–response relationship, with increases in moderate-to-vigorous physical activity, caloric expenditure, and step count associated with shorter Trail Making Test completion time, while increases in light physical activity and caloric expenditure were associated with higher accuracy rates (3). A meta-analysis by Scott et al. (42) confirmed that acute exercise has a small improvement effect on executive function, with moderate evidence strength supporting this conclusion. An acute exercise study targeting young adults with obesity found that N2 amplitude after moderate-intensity aerobic exercise was significantly greater than that in the sedentary control group, indicating that moderate-intensity exercise improves conflict monitoring ability by enhancing attentional resource recruitment (43). From a theoretical mechanism perspective, exercise-induced catecholamine release and increased brain-derived neurotrophic factor (BDNF) are considered important molecular bases for inhibitory control improvement (13, 14, 44). Erickson et al. (15) pointed out that regular exercise can enhance executive function by increasing the functional and structural plasticity of the prefrontal cortex. In summary, acute and long-term exercise have similar effect sizes on inhibitory control but differ in their onset speed and duration of effects. This finding suggests that the choice of exercise modality depends on the intervention goal: acute exercise is suitable for rapid improvement, whereas regular long-term training is necessary for sustained effects.

### 4.2 Effects of exercise interventions on working memory in college students

This study found that long-term exercise intervention significantly improves working memory in college students, whereas the improvement following acute exercise did not reach statistical significance, suggesting that enhancing working memory requires a longer duration of exercise stimulation. From a neural mechanism perspective, regular exercise may enhance working memory by promoting hippocampal neurogenesis and increasing synaptic plasticity (13, 14). In contrast, although a single session of acute exercise can briefly increase hippocampal blood flow, it is insufficient to induce lasting structural changes (7, 15). An ALE meta-analysis of healthy populations found that brain activation during working memory tasks after exercise interventions significantly converged in the right thalamus and right paracentral lobule, suggesting that these regions may be key neural targets through which exercise improves working memory (38). Sandström et al. (45) conducted an RCT evaluating the effects of supramaximal high-intensity interval training (HIT) on working memory in sedentary older adults. Although there was no significant between-group difference in working memory improvement between the HIT group and the moderate-intensity training group, within-group analysis of the HIT group showed a significant improvement in working memory composite scores. More importantly, changes in leg strength significantly predicted increased activation in the right dorsolateral prefrontal cortex (DLPFC), and increased DLPFC activation in turn predicted improved working memory task performance (45).

A study of women with methamphetamine use disorder also provided strong evidence for the differential effects of exercise types on working memory. A 10-week RCT compared the effects of cycle ergometer training and aerobic dance training on spatial and verbal working memory updating (46). The study found that aerobic dance training improved spatial working memory more than verbal working memory, manifested by reduced reaction time and increased right DLPFC activity (46). A study of patients with multiple sclerosis found that 10 weeks of aerobic exercise combined with cognitive tasks, compared with aerobic exercise alone, more significantly increased BDNF levels, dual-task performance, and emotional state, suggesting that the “cognitive-motor dual-task training” paradigm may enhance working memory benefits by increasing neural resource recruitment in the prefrontal cortex (47). A network meta-analysis of children and adolescents found that dance had the best effect on working memory accuracy, while aerobic exercise had the best effect on reaction time (48). A meta-analysis by Haverkamp et al. (9) found that long-term exercise interventions had significant effects on working memory improvement.

### 4.3 Effects of exercise interventions on cognitive flexibility in college students

. The present study found that acute exercise significantly improved cognitive flexibility in college students, whereas long-term exercise showed a small and non-significant effect. From a neurobiological perspective, cognitive flexibility involves the dynamic reconfiguration of the prefrontal-parietal network and may be more sensitive to the transient neurochemical changes induced by acute exercise. A Bayesian meta-analysis by Garrett et al. (49) confirmed that acute exercise has a small beneficial effect on cognition, with the largest effect size observed when cognitive tests were administered immediately after exercise. A dose–response meta-analysis by Pan et al. (50) further demonstrated that cognitive flexibility required the highest acute exercise dose to achieve optimal effects, suggesting that its “threshold” for responding to exercise stimulation is higher than that of inhibitory control and working memory. From a neuroelectrophysiological perspective, acute exercise can enhance P3 and N2 amplitudes, which are closely associated with attentional resource allocation and conflict monitoring (41, 43).

The findings of the present study are inconsistent with those of some meta-analyses in children and adolescents, which reported that acute exercise had no significant effect on cognitive flexibility (41). This discrepancy may be attributed to differences in sample characteristics (college students vs. children and adolescents), exercise protocols, or measurement paradigms. Moreover, an ALE meta-analysis revealed that the number of studies on cognitive flexibility tasks was limited, making it difficult to form a stable pattern of activation convergence, which reflects that this cognitive domain involves a broader prefrontal-parietal network, and that substantial heterogeneity exists across studies (38). The non-significant effect of long-term exercise on cognitive flexibility in the present study may be related to the following factors: (1) most of the included long-term exercise studies predominantly employed closed-skill exercises (e.g., running, swimming) with limited cognitive engagement, whereas improvements in cognitive flexibility may require exercise types with higher cognitive demands, such as open-skill exercises (51, 52); (2) the effects of long-term exercise on the prefrontal-parietal network may be more reflected in enhanced neural efficiency rather than significant changes in behavioral performance.

Furthermore, sleep quality, moderate-to-vigorous physical activity, and cardiorespiratory fitness have complex joint effects on executive function. One study found that moderate-to-vigorous physical activity had a protective effect on working memory in adolescents with poor sleep quality, suggesting that physical activity may indirectly enhance cognitive function by improving sleep quality (53). A narrative review by Ferri et al. (44) also noted that adequate sleep and regular physical activity have independent positive effects on students’ executive function. A Bayesian dose–response meta-analysis by Sui et al. (54) revealed that the improvement of executive function through exercise interventions follows a non-linear relationship. The unity and diversity theory of executive functions proposed by Miyake et al. (55) provides a theoretical framework for understanding the differential responses of various subcomponents to exercise.

### 4.4 Selective effects of exercise dosage on executive function in college students: implications of subgroup analyses

Synthesizing the subgroup analysis results, the present study found that different executive function subcomponents exhibit distinct response patterns to exercise dosage. Regarding session duration, subgroup analyses of long-term interventions revealed that exercise protocols with a session duration of ≥30 min had larger effect sizes on both inhibitory control and cognitive flexibility than those with <30 min, suggesting that 30 min may be the minimum effective duration for producing significant cognitive benefits. Studies in college students have shown that a 15-min acute cycling session can significantly improve executive function (56), and that acute exercise with high physical or high cognitive demands produces more pronounced improvements in inhibitory control (28). In terms of exercise frequency and intervention duration, an 8-week HIIT intervention (three sessions per week) significantly improved physical fitness and cognitive function in sedentary college students (57); a 12-week exercise intervention modulated emotion regulation strategies and enhanced cognitive control in highly stressed college students (58).

Regarding exercise intensity, subgroup analyses of acute exercise showed that high-intensity exercise had larger effect sizes on both inhibitory control and cognitive flexibility than light-to-moderate intensity exercise. A study in sedentary college students found that a 4-min acute Tabata training session significantly improved cognitive performance and increased prefrontal cortex activation (37). The Bayesian dose–response meta-analysis by Pan et al. proposed a “dose-domain-modality” framework, quantifying the dose effects of exercise on executive function: in acute interventions, cognitive flexibility required the highest dose (270 METs), whereas inhibitory control required the lowest dose (130 METs) (50).

Regarding exercise type, subgroup analyses of long-term interventions showed that aerobic exercise significantly improved both inhibitory control and working memory; resistance training showed a large effect on inhibitory control, but this was based on only one study. Notably, for cognitive flexibility, no exercise type reached statistical significance. A Bayesian network meta-analysis in children and adolescents demonstrated an inverted U-shaped dose–response relationship between exercise volume and executive function, with a moderate dose (approximately 1,000 MET-min/week) being most effective, and skill/coordination-based exercises producing significant effects at lower doses (59). Synthesizing the subgroup analyses, inhibitory control responded well to multiple dosage parameters; working memory exhibited a clear “long-term dependence” characteristic; and cognitive flexibility was more sensitive to acute high-intensity exercise. The heterogeneity observed in the present study (long-term cognitive flexibility: *I*^2^ = 60.3%) may be partly explained by differences in measurement paradigms. Furthermore, subgroup analyses indicated that the More-odd shifting task was more sensitive to exercise interventions, whereas the Trail Making Test and Wisconsin Card Sorting Test did not reach statistical significance. In addition, differences in exercise dosage (session duration, intervention duration, exercise intensity) may also contribute to the observed heterogeneity. It should be noted that some subgroup analyses included a limited number of studies (e.g., resistance training, *n* = 1; acute working memory subgroup, *n* = 3); therefore, these findings should be considered exploratory and require confirmation in future high-quality studies.

## 5 Limitations

Several limitations of this study should be considered when interpreting the results. First, the exercise intervention protocols of the included studies were considerably heterogeneous, including differences in exercise type, intensity, frequency, and duration. Second, due to the nature of exercise interventions, participants could not be blinded to group assignment, which may introduce performance bias. Third, this study did not include neuroimaging or neuroelectrophysiological indicators, limiting the in-depth understanding of the mechanisms by which exercise improves executive function. Fourth, this study only included literature published in Chinese and English, which may introduce language bias. Fifth, the number of studies in some subgroup analyses was limited, which may affect statistical power and the robustness of the results. Finally, although we conducted exploratory subgroup analyses by exercise type, the number of studies in some categories (e.g., resistance training, *n* = 1; Tai Chi, *n* = 2; Tabata, *n* = 1) was very limited. Therefore, these findings are hypothesis-generating and require confirmation in future studies with larger sample sizes.

## 6 Conclusion

Based on a systematic review and meta-analysis of 16 randomized controlled trials, the present study demonstrates that acute and long-term exercise have distinct patterns of improvement on executive function in college students. Acute exercise significantly improved cognitive flexibility and inhibitory control but had a limited effect on working memory; long-term exercise significantly improved inhibitory control and working memory but showed no significant effect on cognitive flexibility. Practical implications: For rapid improvement of cognitive flexibility, a single session of acute high-intensity exercise may be preferred; for sustained improvement in working memory, regular long-term exercise should be adopted. Exercise intervention is a safe and effective approach for improving executive function in college students. Future research should further optimize measurement consistency and methodological rigor.

## Data Availability

The original contributions presented in the study are included in the article/Supplementary material, further inquiries can be directed to the corresponding author.
