# Supplementary material for: Effects of exercise intervention on executive function in college students: a systematic review and meta-analysis of randomized controlled trials
Source: Front Public Health. 2026 Jul 13;14:1876948. doi: 10.3389/fpubh.2026.1876948 (PMC13402466; doi:10.3389/fpubh.2026.1876948)
Supplement: Supplementary file 1 [file Data_Sheet_1.docx]

Supplementary Material.

## **1 Supplementary Tables**

**Table S1.** The preferred reporting items for systematic reviews and meta-analysis (PRISMA) 2020 main checklist.

| **Topic** | **No.** | **Item** | **Location where item is reported** |
| --- | --- | --- | --- |
| **TITLE** |  |  |  |
| **Title** | 1 | Identify the report as a systematic review. | Title |
| **ABSTRACT** |  |  |  |
| **Abstract** | 2 | See the PRISMA 2020 for Abstracts checklist | Abstract |
| **INTRODUCTION** |  |  |  |
| **Rationale** | 3 | Describe the rationale for the review in the context of existing knowledge. | 1 Introduction |
| **Objectives** | 4 | Provide an explicit statement of the objective(s) or question(s) the review addresses. | 1 Introduction |
| **METHODS** |  |  |  |
| **Eligibility criteria** | 5 | Specify the inclusion and exclusion criteria for the review and how studies were grouped for the syntheses. | 2.3 Inclusion and exclusion criteria |
| **Information sources** | 6 | Specify all databases, registers, websites, organizations, reference lists and other sources searched or consulted to identify studies. Specify the date when each source was last searched or consulted. | 2.2 Search strategy |
| **Search strategy** | 7 | Present the full search strategies for all databases, registers and websites, including any filters and limits used. | 2.2 Search strategy |
| **Selection process** | 8 | Specify the methods used to decide whether a study met the inclusion criteria of the review, including how many reviewers screened each record and each report retrieved, whether they worked independently, and if applicable, details of automation tools used in the process. | 2.4 Literature screening and data extraction |
| **Data collection process** | 9 | Specify the methods used to collect data from reports, including how many reviewers collected data from each report, whether they worked independently, any processes for obtaining or confirming data from study investigators, and if applicable, details of automation tools used in the process. | 2.4 Literature screening and data extraction |
| **Data items** | 10a | List and define all outcomes for which data were sought. Specify whether all results that were compatible with each outcome domain in each study were sought (e.g. for all measures, time points, analyses), and if not, the methods used to decide which results to collect. | 2.3 Inclusion and exclusion criteria  2.4 Literature screening and data extraction |
|  | 10b | List and define all other variables for which data were sought (e.g. participant and intervention characteristics, funding sources). Describe any assumptions made about any missing or unclear information. | 2.4 Literature screening and data extraction |
| **Study risk of bias assessment** | 11 | Specify the methods used to assess risk of bias in the included studies, including details of the tool(s) used, how many reviewers assessed each study and whether they worked independently, and if applicable, details of automation tools used in the process. | 2.5 Risk of bias assessment |
| **Effect measures** | 12 | Specify for each outcome the effect measure(s) (e.g. risk ratio, mean difference) used in the synthesis or presentation of results. | 2.6 Statistical analysis |
| **Synthesis methods** | 13a | Describe the processes used to decide which studies were eligible for each synthesis (e.g. tabulating the study intervention characteristics and comparing against the planned groups for each synthesis (item 5)). | Table 1 Characteristics of studies included  Table 2 Exercise characteristics of included studies |
|  | 13b | Describe any methods required to prepare the data for presentation or synthesis, such as handling of missing summary statistics, or data conversions. | 2.6 Statistical analysis |
|  | 13c | Describe any methods used to tabulate or visually display results of individual studies and syntheses. | Table 1 Characteristics of studies included  Table 2 Exercise characteristics of included studies |
|  | 13d | Describe any methods used to synthesize results and provide a rationale for the choice(s). If meta-analysis was performed, describe the model(s), method(s) to identify the presence and extent of statistical heterogeneity, and software package(s) used. | 2.6 Statistical analysis |
|  | 13e | Describe any methods used to explore possible causes of heterogeneity among study results (e.g. subgroup analysis, meta-regression). | 2.7 Subgroup analysis |
|  | 13f | Describe any sensitivity analyses conducted to assess robustness of the synthesized results. | 2.9 Sensitivity analysis |
| **Reporting bias assessment** | 14 | Describe any methods used to assess risk of bias due to missing results in a synthesis (arising from reporting biases). | 2.5 Risk of bias assessment |
| **Certainty assessment** | 15 | Describe any methods used to assess certainty (or confidence) in the body of evidence for an outcome. | — |
| **RESULTS** |  |  |  |
| **Study selection** | 16a | Describe the results of the search and selection process, from the number of records identified in the search to the number of studies included in the review, ideally using a flow diagram. | 3.1 Study selection  Figure 1  PRISMA study flow diagram |
|  | 16b | Cite studies that might appear to meet the inclusion criteria, but which were excluded, and explain why they were excluded. | 3.1 Study selection  Figure 1  PRISMA study flow diagram |
| **Study characteristics** | 17 | Cite each included study and present its characteristics. | 3.2 Characteristics of included studies |
| **Risk of bias in studies** | 18 | Present assessments of risk of bias for each included study. | 3.3 Risk of bias |
| **Results of individual studies** | 19 | For all outcomes, present, for each study: (a) summary statistics for each group (where appropriate) and (b) an effect estimates and its precision (e.g. confidence/credible interval), ideally using structured tables or plots. | 3.4 Meta-analysis results  3.5 Subgroup analysis |
| **Results of syntheses** | 20a | For each synthesis, briefly summaries the characteristics and risk of bias among contributing studies. | 3.3 Risk of bias  3.4 Meta-analysis results  3.5 Subgroup analysis |
|  | 20b | Present results of all statistical syntheses conducted. If meta-analysis was done, present for each the summary estimate and its precision (e.g. confidence/credible interval) and measures of statistical heterogeneity. If comparing groups, describe the direction of the effect. | 3.4 Meta-analysis results  3.5 Subgroup analysis |
|  | 20c | Present results of all investigations of possible causes of heterogeneity among study results. | 3.5 Subgroup analysis |
|  | 20d | Present results of all sensitivity analyses conducted to assess the robustness of the synthesized results. | 3.7 Sensitivity analysis |
| **Reporting biases** | 21 | Present assessments of risk of bias due to missing results (arising from reporting biases) for each synthesis assessed. | - |
| **Certainty of evidence** | 22 | Present assessments of certainty (or confidence) in the body of evidence for each outcome assessed. | - |
| **DISCUSSION** |  |  |  |
| **Discussion** | 23a | Provide a general interpretation of the results in the context of other evidence. | 4 Discussion |
|  | 23b | Discuss any limitations of the evidence included in the review. | 4 Discussion |
|  | 23c | Discuss any limitations of the review processes used. | 4 Discussion |
|  | 23d | Discuss implications of the results for practice, policy, and future research. | 4 Discussion |
| **OTHER INFORMATION** |  |  |  |
| **Registration and protocol** | 24a | Provide registration information for the review, including register name and registration number, or state that the review was not registered. | 2.1 Protocol and registration |
|  | 24b | Indicate where the review protocol can be accessed, or state that a protocol was not prepared. | 2.1 Protocol and registration |
|  | 24c | Describe and explain any amendments to information provided at registration or in the protocol. | 2.1 Protocol and registration |
| **Support** | 25 | Describe sources of financial or non-financial support for the review, and the role of the funders or sponsors in the review. | Funding information |
| **Competing interests** | 26 | Declare any competing interests of review authors. | Competing interests |
| **Availability of data, code and other materials** | 27 | Report which of the following are publicly available and where they can be found template data collection forms; data extracted from included studies; data used for all analyses; analytic code; any other materials used in the review. | Data availability |

**Table S2.** Database search strategy.

| **Database** | Search strategies |
| --- | --- |
| **PubMed** | **#1** "Exercise"[Mesh] OR "Physical Activity"[Mesh] OR "Resistance Training"[Mesh] OR "High-Intensity Interval Training"[Mesh] OR "Tai Ji"[Mesh] OR "Yoga"[Mesh] OR "Running"[Mesh] OR "Swimming"[Mesh] OR "Sports"[Mesh] OR exercise[Title/Abstract] OR physical activity[Title/Abstract] OR aerobic[Title/Abstract] OR resistance training[Title/Abstract] OR strength training[Title/Abstract] OR HIIT[Title/Abstract] OR high-intensity interval training[Title/Abstract] OR tai chi[Title/Abstract] OR yoga[Title/Abstract] OR running[Title/Abstract] OR swimming[Title/Abstract] OR jumping[Title/Abstract] OR Tabata[Title/Abstract]  **#2** "Students"[Mesh] OR "Universities"[Mesh] OR college student[Title/Abstract] OR university student[Title/Abstract] OR undergraduate[Title/Abstract] OR young adult[Title/Abstract] OR college[Title/Abstract] OR campus[Title/Abstract]  **#3** "Executive Function"[Mesh] OR executive function[Title/Abstract] OR inhibitory control[Title/Abstract] OR working memory[Title/Abstract] OR cognitive flexibility[Title/Abstract] OR Stroop[Title/Abstract] OR Flanker[Title/Abstract] OR Go/No-Go[Title/Abstract] OR N-back[Title/Abstract] OR task switching[Title/Abstract] OR Trail Making Test[Title/Abstract] OR Wisconsin Card Sorting[Title/Abstract]  **#4** "Randomized Controlled Trial"[Publication Type] OR randomized controlled trial[Title/Abstract] OR RCT[Title/Abstract] OR randomised[Title/Abstract] OR randomized[Title/Abstract] OR controlled trial[Title/Abstract]  **#5 #1 AND #2 AND #3 AND #4** |
| **Embase** | **#1** 'exercise'/exp OR 'physical activity'/exp OR 'resistance training'/exp OR 'high intensity interval training'/exp OR 'tai chi'/exp OR 'yoga'/exp OR 'running'/exp OR 'swimming'/exp OR exercise:ab,ti OR 'physical activity':ab,ti OR aerobic:ab,ti OR 'resistance training':ab,ti OR HIIT:ab,ti OR 'tai chi':ab,ti OR yoga:ab,ti  **#2** 'student'/exp OR 'university student'/exp OR 'college student':ab,ti OR 'university student':ab,ti OR undergraduate:ab,ti OR 'young adult':ab,ti  **#3** 'executive function'/exp OR 'executive function':ab,ti OR 'inhibitory control':ab,ti OR 'working memory':ab,ti OR 'cognitive flexibility':ab,ti OR stroop:ab,ti OR flanker:ab,ti OR 'n-back':ab,ti OR 'trail making test':ab,ti  **#4** 'randomized controlled trial'/exp OR rct:ab,ti OR randomized:ab,ti OR randomised:ab,ti  **#5 #1 AND #2 AND #3 AND #4** |
| **Cochrane library** | **#1** MeSH descriptor: [Exercise] explode all trees OR MeSH descriptor: [Physical Activity] explode all trees OR MeSH descriptor: [Resistance Training] explode all trees OR MeSH descriptor: [High-Intensity Interval Training] explode all trees OR MeSH descriptor: [Tai Ji] explode all trees OR MeSH descriptor: [Yoga] explode all trees OR (exercise OR physical activity OR aerobic OR resistance training OR HIIT OR tai chi OR yoga OR running OR swimming):ti,ab,kw  **#2** MeSH descriptor: [Students] explode all trees OR MeSH descriptor: [Universities] explode all trees OR (college student OR university student OR undergraduate OR young adult):ti,ab,kw  **#3** MeSH descriptor: [Executive Function] explode all trees OR (executive function OR inhibitory control OR working memory OR cognitive flexibility OR Stroop OR Flanker OR N-back OR task switching):ti,ab,kw  **#4** MeSH descriptor: [Randomized Controlled Trial] explode all trees OR (randomized OR randomised OR RCT OR controlled trial):ti,ab,kw  **#5 #1 AND #2 AND #3 AND #4** |
| **Web of Science** | **#1** TS=(exercise OR "physical activity" OR aerobic OR "resistance training" OR "strength training" OR HIIT OR "high-intensity interval training" OR "tai chi" OR yoga OR running OR swimming OR jumping OR Tabata)  **#2** TS=("college student" OR "university student" OR undergraduate OR "young adult" OR college OR campus)  **#3** TS=("executive function" OR "inhibitory control" OR "working memory" OR "cognitive flexibility" OR Stroop OR Flanker OR "Go/No-Go" OR "N-back" OR "task switching" OR "Trail Making Test" OR "Wisconsin Card Sorting")  **#4** TS=("randomized controlled trial" OR RCT OR randomized OR randomised OR "controlled trial")  **#5 #1 AND #2 AND #3 AND #4** |
| **Scopus** | **#1** TITLE-ABS-KEY(exercise OR "physical activity" OR aerobic OR "resistance training" OR HIIT OR "high-intensity interval training" OR "tai chi" OR yoga OR running OR swimming OR jumping OR Tabata)  **#2** TITLE-ABS-KEY("college student" OR "university student" OR undergraduate OR "young adult")  **#3** TITLE-ABS-KEY("executive function" OR "inhibitory control" OR "working memory" OR "cognitive flexibility" OR stroop OR flanker OR "go/no-go" OR "n-back" OR "task switching" OR "trail making test")  **#4** TITLE-ABS-KEY("randomized controlled trial" OR rct OR randomized OR randomised)  **#5 #1 AND #2 AND #3 AND #4** |
| **China National Knowledge Infrastructure (CNKI)** | (SU = '运动' OR '体育锻炼' OR '有氧运动' OR '抗阻训练' OR '高强度间歇训练' OR 'HIIT' OR '太极拳' OR '瑜伽' OR '跑步' OR '游泳' OR '跳跃' OR 'Tabata')  AND  (SU = '大学生' OR '高校学生' OR '本科生')  AND  (SU = '执行功能' OR '抑制控制' OR '工作记忆' OR '认知灵活性' OR 'Stroop' OR 'Flanker' OR 'Go/No-Go' OR 'N-back' OR '任务切换' OR '连线测验' OR '威斯康星卡片分类')  AND  (SU = '随机对照试验' OR 'RCT') |
| **Wanfang** | 主题:(运动 OR 体育锻炼 OR 有氧运动 OR 抗阻训练 OR 高强度间歇训练 OR HIIT OR 太极拳 OR 瑜伽)  AND 主题:(大学生 OR 高校学生)  AND 主题:(执行功能 OR 抑制控制 OR 工作记忆 OR 认知灵活性 OR Stroop OR Flanker OR N-back)  AND 主题:(随机对照试验 OR RCT) |

**Table S3.** Exercise Intensity Classification Standards.

| Intensity Level | %HRmax | %HRR | RPE (6-20 Borg) | RPE (CR-10) | RM |
| --- | --- | --- | --- | --- | --- |
| Low | <57% | <40% | <11 | <3 | >15 RM |
| Moderate | 57%-74% | 40%-69% | 11-13 | 3-5 | 8-15 RM |
| High | ≥75% | ≥70% | ≥14 | ≥6 | ≤7 RM |

**Table S4.** Subgroup analysis of the effects of acute exercise interventions on inhibitory control.

| Subgroup | Number of studies | Number of Effect Sizes | *I^2^* | *P* | Hedges’ g | 95%CI |
| --- | --- | --- | --- | --- | --- | --- |
| Measurement paradigm | | | | | | |
| Stroop color-word test | 3 | 4 | 69.0% | 0.021 | 0.41 | (-0.23, 1.05) |
| Go/NoGo task | 1 | 2 | 7.0% | 0.300 | 0.94 | (0.35, 1.53) |
| Antisaccade Task | 1 | 1 | - | - | 0.22 | (-0.48, 0.91) |
| Exercise type | | | | | | |
| High-intensity functional training | 1 | 1 | 0.0% | <0.001 | 0.50 | (-0.34, 1.35) |
| Aerobic exercise | 4 | 5 | 70.4% | 0.009 | 0.48 | (-0.11, 1.07) |
| Tabata training | 1 | 1 | - | - | 0.79 | (0.09, 1.50) |
| Session time | | | | | | |
| <30 min | 4 | 5 | 70.6% | 0.009 | 0.47 | (-0.10, 1.04) |
| ≥30 min | 1 | 2 | 0.0% | 0.539 | 0.69 | (0.09, 1.29) |
| Exercise intensity | | | | | | |
| Light-moderate | 4 | 4 | 76.3% | 0.005 | 0.45 | (-0.27, 1.18) |
| High | 3 | 3 | 0.0% | 0.873 | 0.67 | (0.23, 1.11) |

**Table S5.** Subgroup analysis of the effects of long-term exercise interventions on inhibitory control.

| Subgroup | Number of studies | Number of Effect Sizes | *I^2^* | *P* | Hedges’ g | 95%CI |
| --- | --- | --- | --- | --- | --- | --- |
| Measurement paradigm | | | | | | |
| Stroop color-word test | 5 | 8 | 8.0% | 0.368 | 0.75 | (0.50, 0.99) |
| Go/NoGo task | 1 | 1 | - | - | 0.17 | (-0.35, 0.69) |
| Flanker task | 3 | 4 | 0.0% | 0.441 | 0.29 | (-0.01, 0.60) |
| Exercise type | | | | | | |
| High-intensity interval training | 2 | 2 | 0.0% | 0.620 | 0.66 | (0.08, 1.24) |
| Aerobic exercise | 5 | 6 | 63.2% | 0.018 | 0.51 | (0.24, 0.79) |
| Resistance exercise | 1 | 1 | - | - | 1.07 | (0.24, 1.90) |
| Jump training | 1 | 1 | - | - | 0.17 | (-0.35, 0.69) |
| Tai Chi | 2 | 2 | 0.0% | 0.856 | 0.54 | (0.20, 0.88) |
| Multimodal exercise | 1 | 1 | - | - | 0.57 | (-0.24, 1.37) |
| Session time | | | | | | |
| <30 min | 3 | 4 | 0.0% | 0.752 | 0.40 | (0.10, 0.71) |
| ≥30 min | 6 | 9 | 48.2% | 0.051 | 0.59 | (0.37, 0.81) |
| Frequency of exercise | | | | | | |
| 3 times/week | 8 | 11 | 19.1% | 0.262 | 0.59 | (0.40, 0.78) |
| 4 times/week | 1 | 2 | 0.0% | 0.624 | -0.05 | (-0.59, 0.48) |
| Exercise intensity | | | | | | |
| Light-moderate | 6 | 7 | 55.2% | 0.037 | 0.62 | (0.38, 0.86) |
| High | 4 | 5 | 0.0% | 0.630 | 0.36 | (0.05, 0.68) |
| Duration of exercise | | | | | | |
| 6 weeks | 1 | 2 | 8.6% | 0.296 | 1.12 | (0.50, 1.74) |
| 8 weeks | 5 | 6 | 38.9% | 0.147 | 0.53 | (0.30, 0.77) |
| 12 weeks | 3 | 5 | 0.0% | 0.450 | 0.37 | (0.07, 0.67) |

**Table S6.** Subgroup analysis of the effects of acute exercise interventions on working memory.

| Subgroup | Number of studies | Number of Effect Sizes | *I^2^* | *P* | Hedges’ g | 95%CI |
| --- | --- | --- | --- | --- | --- | --- |
| Exercise type | | | | | | |
| High-intensity functional training | 1 | 1 | - | - | -0.12 | (-0.95, 0.72) |
| Aerobic exercise | 3 | 4 | 0.0% | 0.654 | 0.14 | (-0.20, 0.48) |
| Session time | | | | | | |
| <30 min | 2 | 3 | 0.0% | 0.702 | 0.07 | (-0.30, 0.44) |
| ≥30 min | 1 | 2 | 8.4% | 0.296 | 0.20 | (-0.39, 0.79) |
| Exercise intensity | | | | | | |
| Light-moderate | 3 | 3 | 0.0% | 0.504 | 0.10 | (-0.28, 0.47) |
| High | 2 | 2 | 0.0% | 0.454 | 0.12 | (-0.44, 0.68) |

**Table S7.** Subgroup analysis of the effects of long-term exercise interventions on working memory.

| Subgroup | Number of studies | Number of Effect Sizes | *I^2^* | *P* | Hedges’ g | 95%CI |
| --- | --- | --- | --- | --- | --- | --- |
| Exercise type | | | | | | |
| High-intensity interval training | 2 | 2 | 4.0% | 0.307 | 0.64 | (0.07, 1.21) |
| Aerobic exercise | 5 | 5 | 0.0% | 0.567 | 0.69 | (0.40, 0.98) |
| Resistance exercise | 1 | 1 | - | - | 0.46 | (0.32, 1.25) |
| Jump training | 1 | 1 | - | - | 0.69 | (0.16, 1.23) |
| Tai Chi | 1 | 1 | - | - | 0.15 | (-0.33, 0.64) |
| Session time | | | | | | |
| <30 min | 1 | 1 | - | - | 0.69 | (0.16, 1.23) |
| ≥30 min | 6 | 9 | 0.0% | 0.480 | 0.55 | (0.33, 0.77) |
| Frequency of exercise | | | | | | |
| 3 times/week | 6 | 8 | 4.5% | 0.396 | 0.58 | (0.36, 0.79) |
| 4 times/week | 1 | 2 | 0.0% | 0.510 | 0.55 | (0.00, 1.10) |
| Exercise intensity | | | | | | |
| Light-moderate | 5 | 6 | 0.0% | 0.666 | 0.66 | (0.39, 0.93) |
| High | 3 | 3 | 0.0% | 0.589 | 0.67 | (0.28, 1.06) |
| Duration of exercise | | | | | | |
| 6 weeks | 1 | 2 | 0.0% | 0.533 | 1.16 | (0.54, 1.78) |
| 8 weeks | 5 | 6 | 0.0% | 0.690 | 0.49 | (0.26, 0.73) |
| 12 weeks | 1 | 2 | 0.0% | 0.510 | 0.55 | (0.00, 1.10) |

**Table S8.** Subgroup analysis of the effects of acute exercise interventions on cognitive flexibility.

| Subgroup | Number of studies | Number of Effect Sizes | *I^2^* | *P* | Hedges’ g | 95%CI |
| --- | --- | --- | --- | --- | --- | --- |
| Measurement paradigm | | | | | | |
| More-odd shifting | 2 | 3 | 72.1% | 0.028 | 0.63 | (0.14, 1.40) |
| Trail making test | 1 | 3 | 0.0% | 0.503 | 1.17 | (0.35, 1.99) |
| Exercise intensity | | | | | | |
| Light-moderate | 3 | 4 | 60.3% | 0.056 | 0.69 | (-0.06, 1.44) |
| High | 2 | 2 | 0.0% | 0.747 | 1.16 | (0.45, 1.86) |

**Table S9.** Subgroup analysis of the effects of long-term exercise interventions on cognitive flexibility.

| Subgroup | Number of studies | Number of Effect Sizes | *I^2^* | *P* | Hedges’ g | 95%CI |
| --- | --- | --- | --- | --- | --- | --- |
| Measurement paradigm | | | | | | |
| More-odd shifting task | 5 | 7 | 34.8% | 0.163 | 0.46 | (0.16, 0.77) |
| Wisconsin Card Sorting Test | 1 | 2 | 77.7% | 0.034 | -0.50 | (-1.49, 0.49) |
| Trail Marking Test | 1 | 1 | - | - | 0.27 | (-0.28, 0.81) |
| Task-Switching paradigms | 1 | 1 | - | - | 0.15 | (-0.37, 0.67) |
| Exercise type | | | | | | |
| High-intensity interval training | 4 | 4 | 78.5% | 0.003 | 0.11 | (-0.65, 0.86) |
| Aerobic exercise | 5 | 5 | 58.4% | 0.047 | 0.39 | (-0.06, 0.84) |
| Jump training | 1 | 1 | - | - | 0.29 | (-0.20, 0.77) |
| Tai Chi | 1 | 1 | - | - | 0.15 | (-0.37, 0.67) |
| Session time | | | | | | |
| <30 min | 3 | 4 | 68.4% | 0.024 | -0.12 | (-0.64, 0.41) |
| ≥30 min | 5 | 7 | 34.8% | 0.163 | 0.46 | (0.16, 0.77) |
| Frequency of exercise | | | | | | |
| 3 times/week | 7 | 9 | 64.9% | 0.004 | 0.19 | (-0.15, 0.52) |
| 4 times/week | 1 | 2 | 0.0% | 0.651 | 0.62 | (0.07, 1.17) |
| Exercise intensity | | | | | | |
| Light-moderate | 4 | 4 | 63.5% | 0.042 | 0.49 | (-0.05, 1.03) |
| High | 5 | 6 | 64.5% | 0.015 | 0.09 | (-0.35, 0.53) |
| Duration of exercise | | | | | | |
| 6 weeks | 1 | 2 | 0.0% | 0.765 | 0.57 | (-0.02, 1.15) |
| 8 weeks | 5 | 5 | 51.5% | 0.083 | 0.30 | (-0.03, 0.64) |
| 12 weeks | 2 | 4 | 77.7% | 0.004 | 0.04 | (-0.72, 0.80) |

## **2 Supplementary Figures**


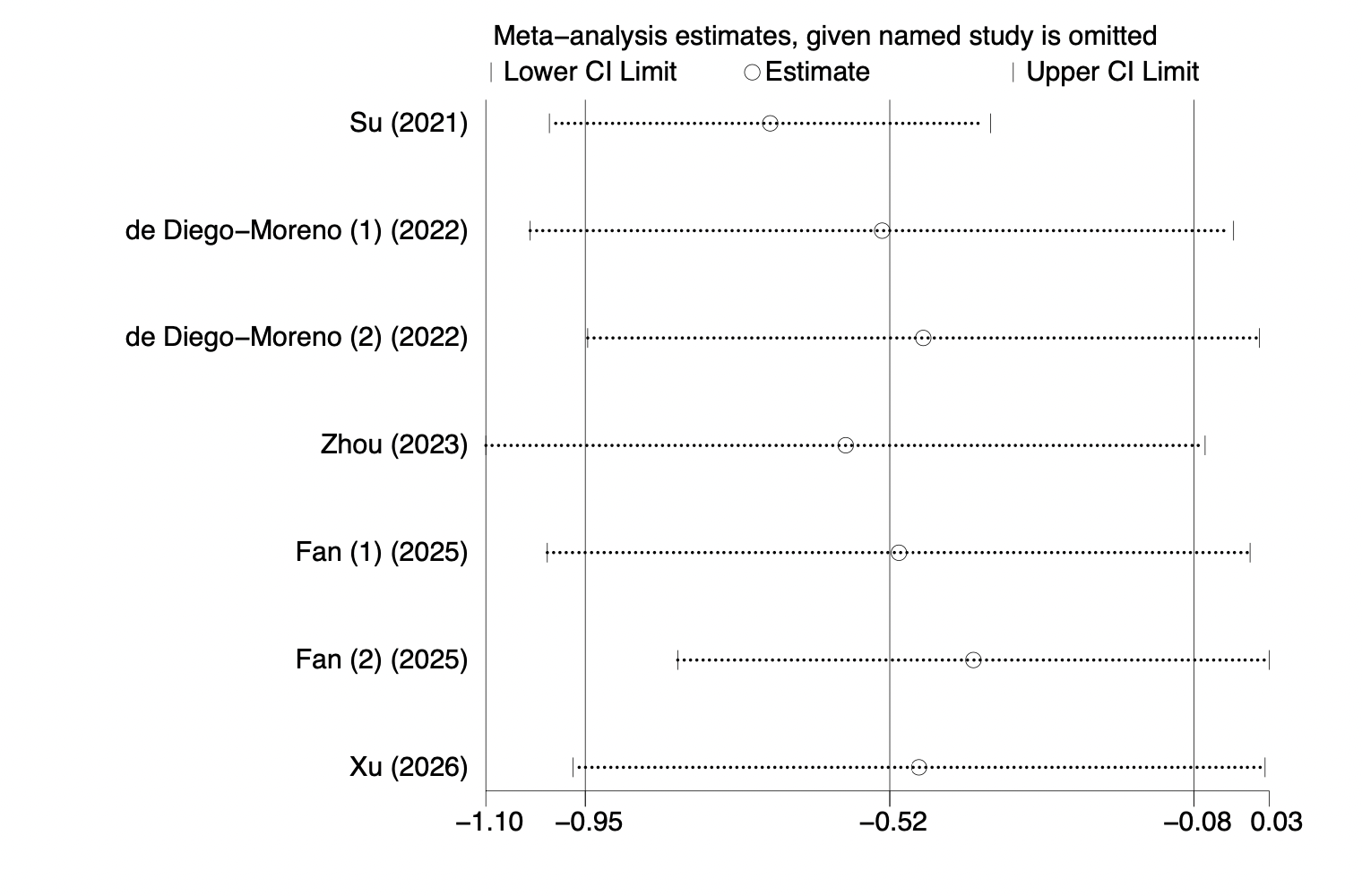


**Figure S1.** Sensitivity analysis of the effects of acute exercise on inhibitory control.


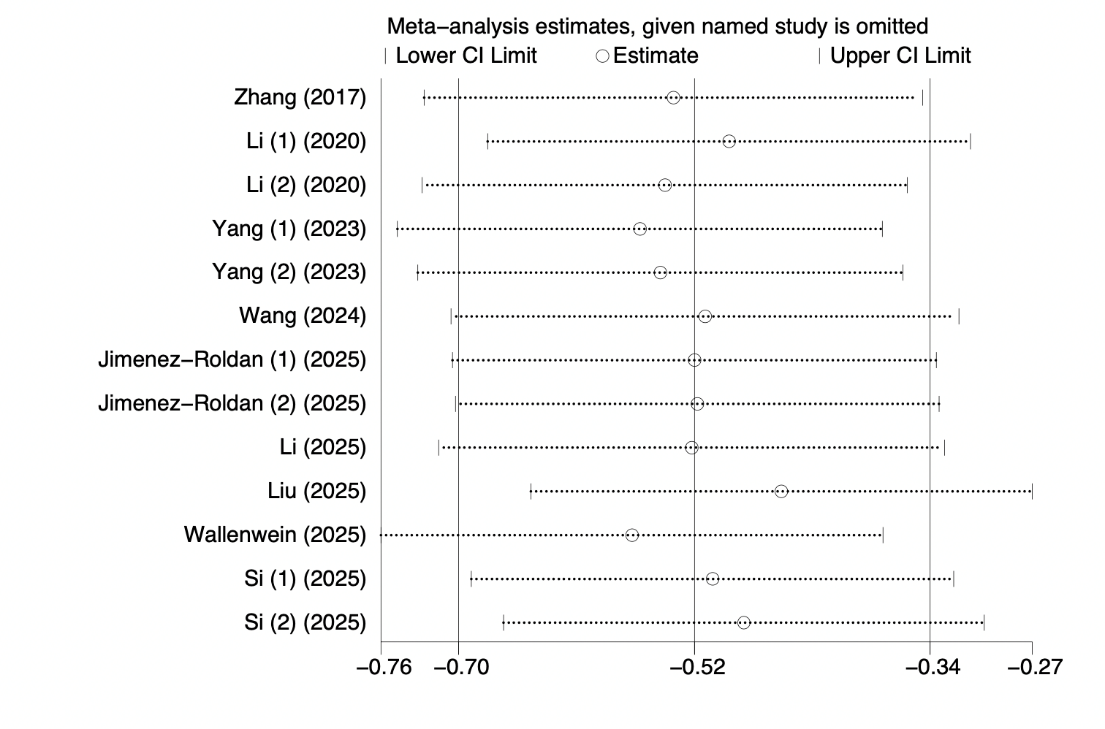


**Figure S2.** Sensitivity analysis of the effects of long-term exercise on inhibitory control.


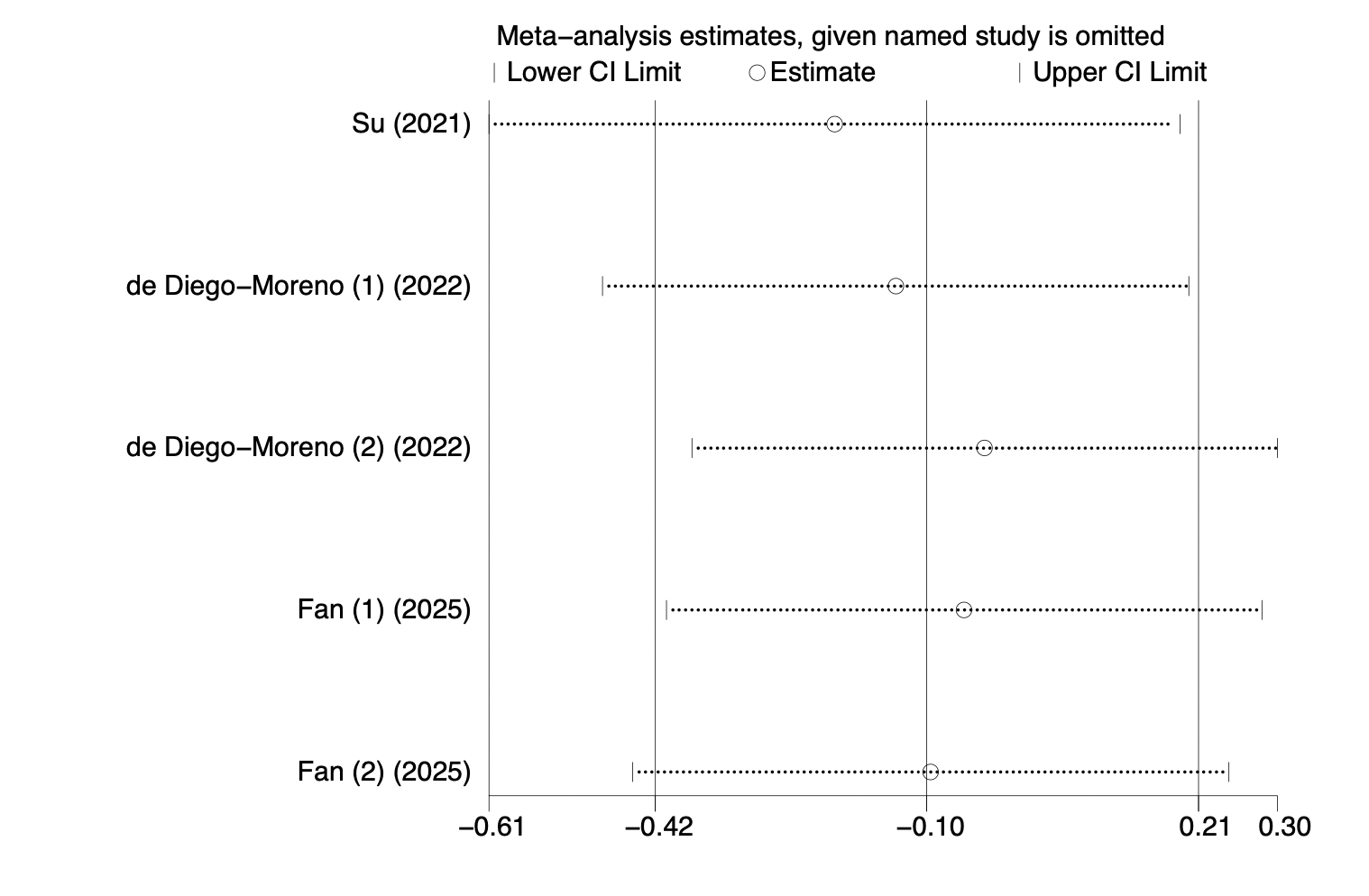


**Figure S3.** Sensitivity analysis of the effects of acute exercise working memory.


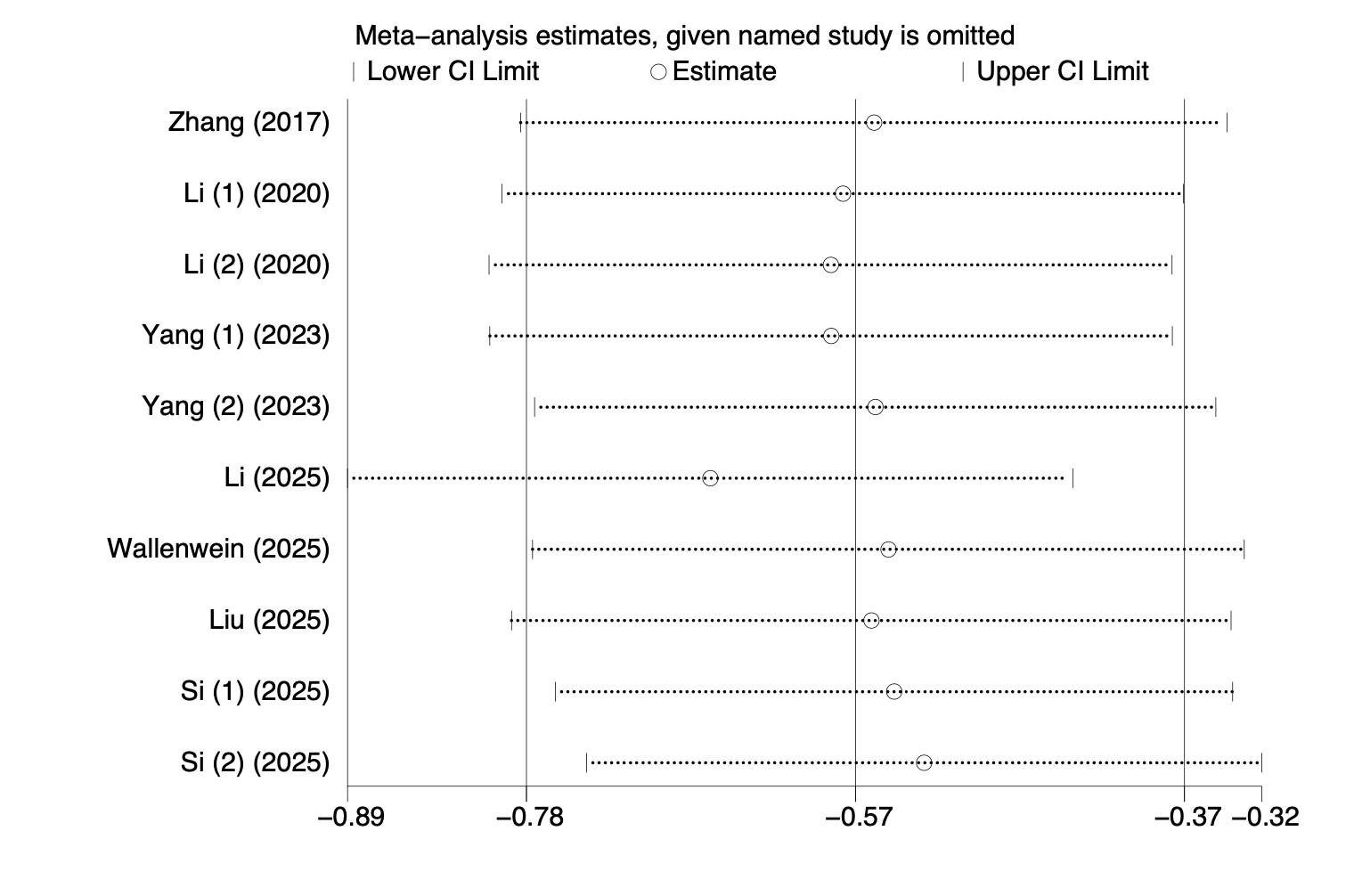


**Figure S4.** Sensitivity analysis of the effects of long-term exercise working memory.


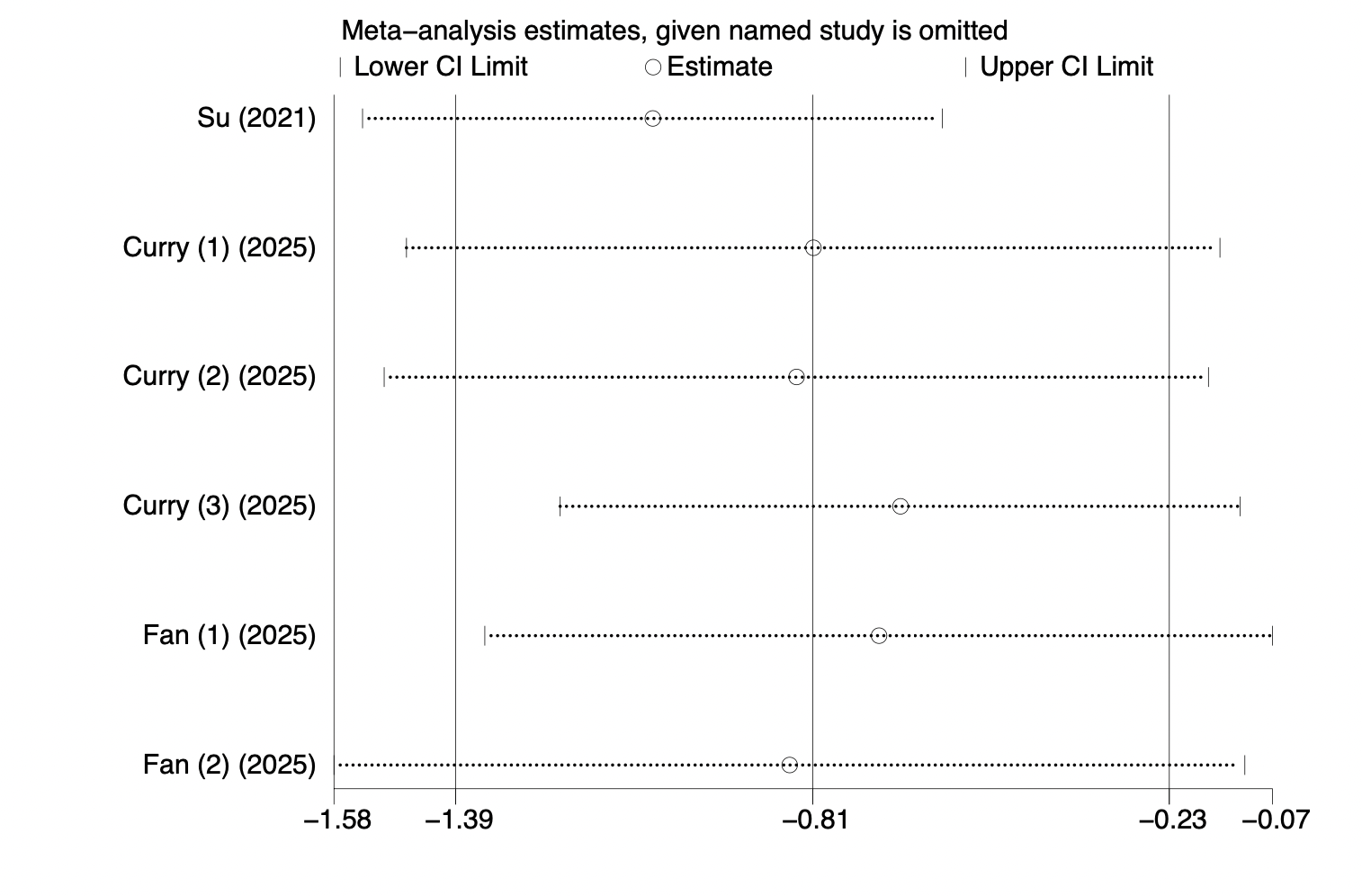


**Figure S5.** Sensitivity analysis of the effects of acute exercise cognitive flexibility.


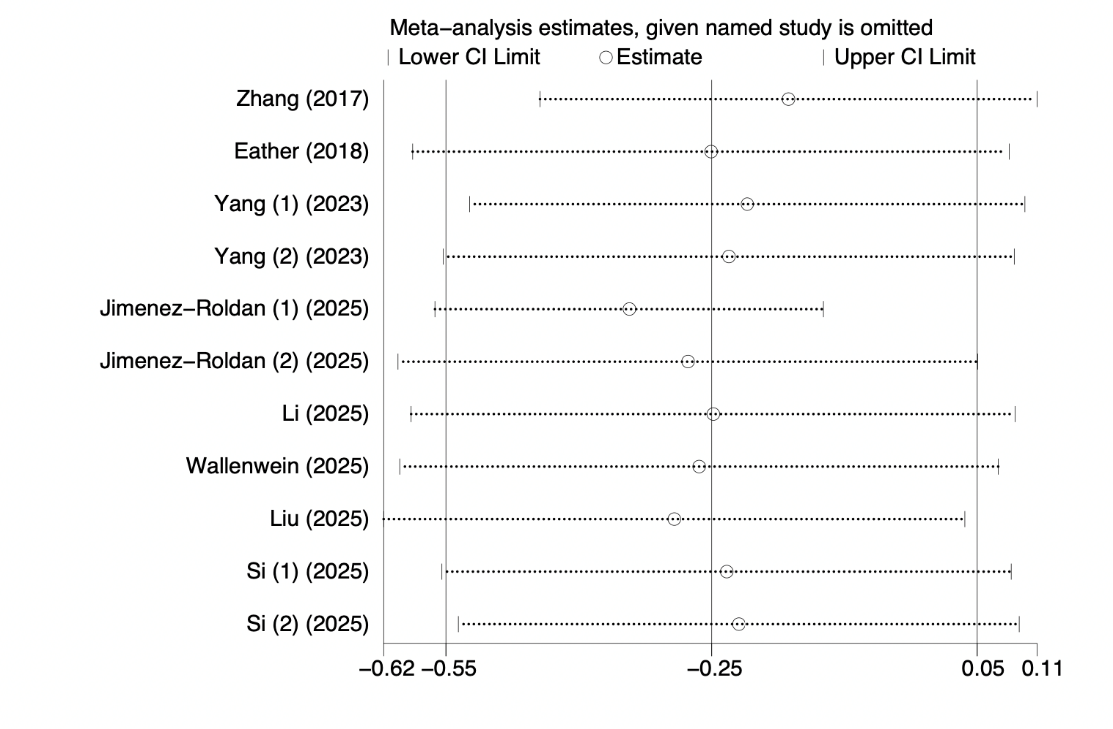


**Figure S6.** Sensitivity analysis of the effects of long-term exercise cognitive flexibility.


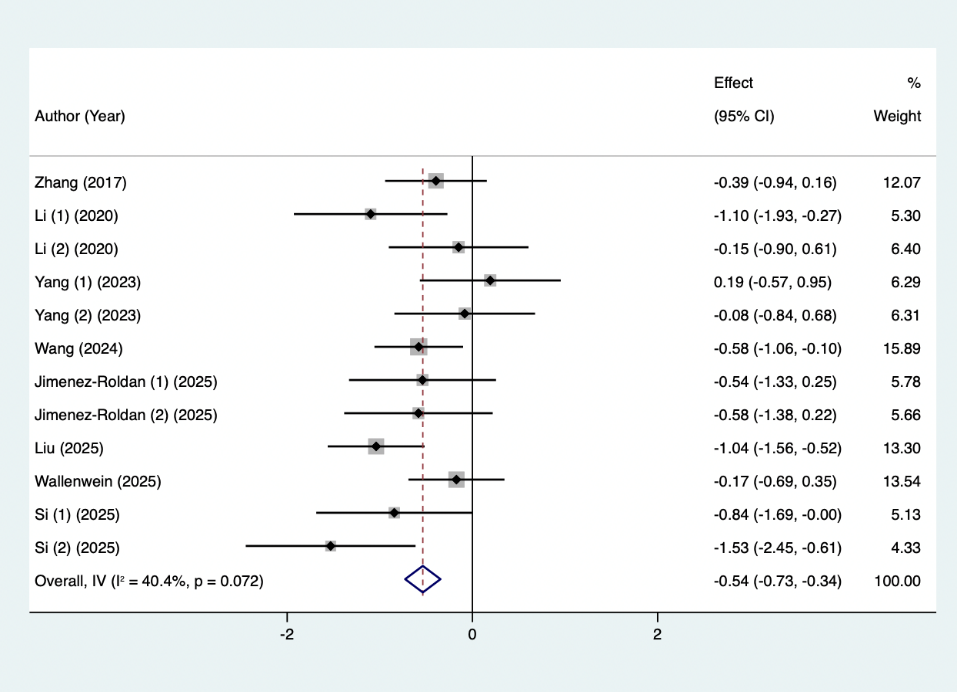


**Figure S7.** Sensitivity analysis for long-term inhibitory control: Pooled effect sizes before and after excluding Li et al. (2025).


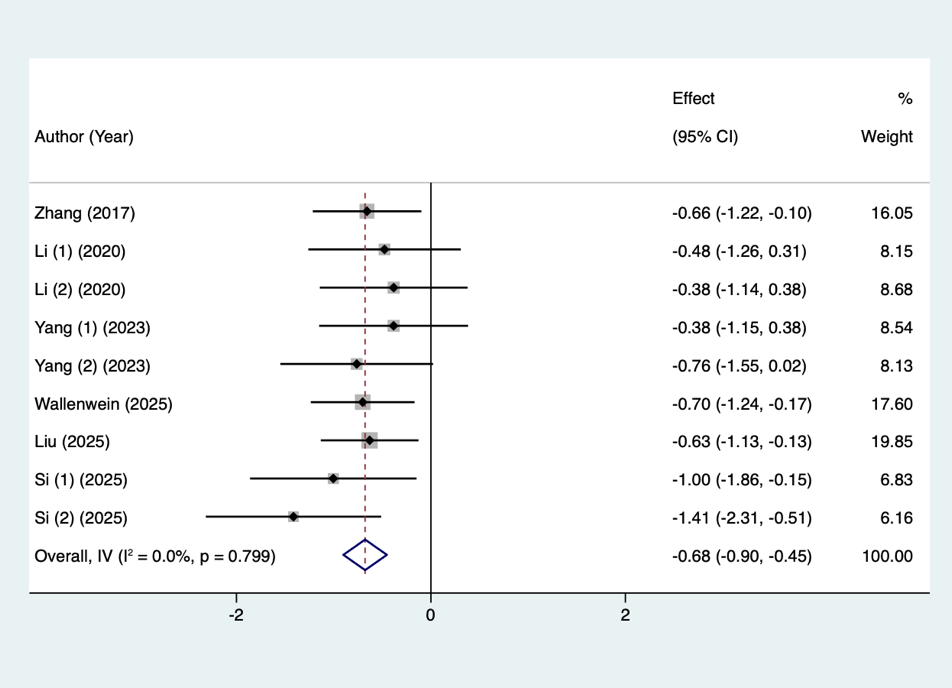


**Figure S8.** Sensitivity analysis for long-term working memory: Pooled effect sizes before and after excluding Li et al. (2025).


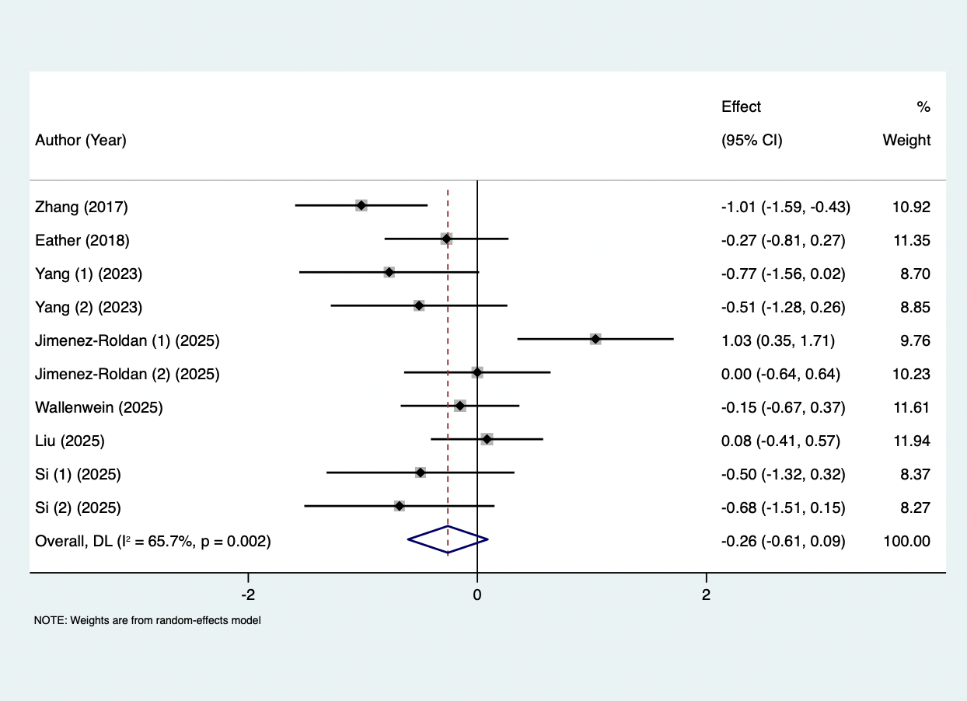


**Figure S9.** Sensitivity analysis for long-term cognitive flexibility: Pooled effect sizes before and after excluding Li et al. (2025).
